# Supplementary material for: Bi-allelic Mutations in NDUFA6 Establish Its Role in Early-Onset Isolated Mitochondrial Complex I Deficiency
Source: Am J Hum Genet. 2018 Sep 20;103(4):592–601. doi: 10.1016/j.ajhg.2018.08.013 (PMC6174280; doi:10.1016/j.ajhg.2018.08.013)
Supplement: Document S1. Figures S1–S6 and Table S1 [file mmc1.pdf]

The American Journal of Human Genetics, Volume *103*

Supplemental Data

Bi-allelic Mutations in *NDUFA6*

Establish Its Role in Early-Onset

Isolated Mitochondrial Complex I Deficiency

Charlotte L. Alston, Juliana Heidler, Marris G. Dibley, Laura S. Kremer, Lucie S. Taylor, Carl Fratter, Courtney E. French, Ruth I.C. Glasgow, René G. Feichtinger, Isabelle Delon, Alistair T. Pagnamenta, Helen Dolling, Hugh Lemonde, Neil Aiton, Alf Bjørnstad, Lisa Henneke, Jutta Gärtner, Holger Thiele, Katerina Tauchmannova, Gerardine Quaghebeur, Josef Houstek, Wolfgang Sperl, F. Lucy Raymond, Holger Prokisch, Johannes A. Mayr, Robert McFarland, Joanna Poulton, Michael T. Ryan, Ilka Wittig, Marco Henneke, and Robert W. Taylor

Supplemental figures

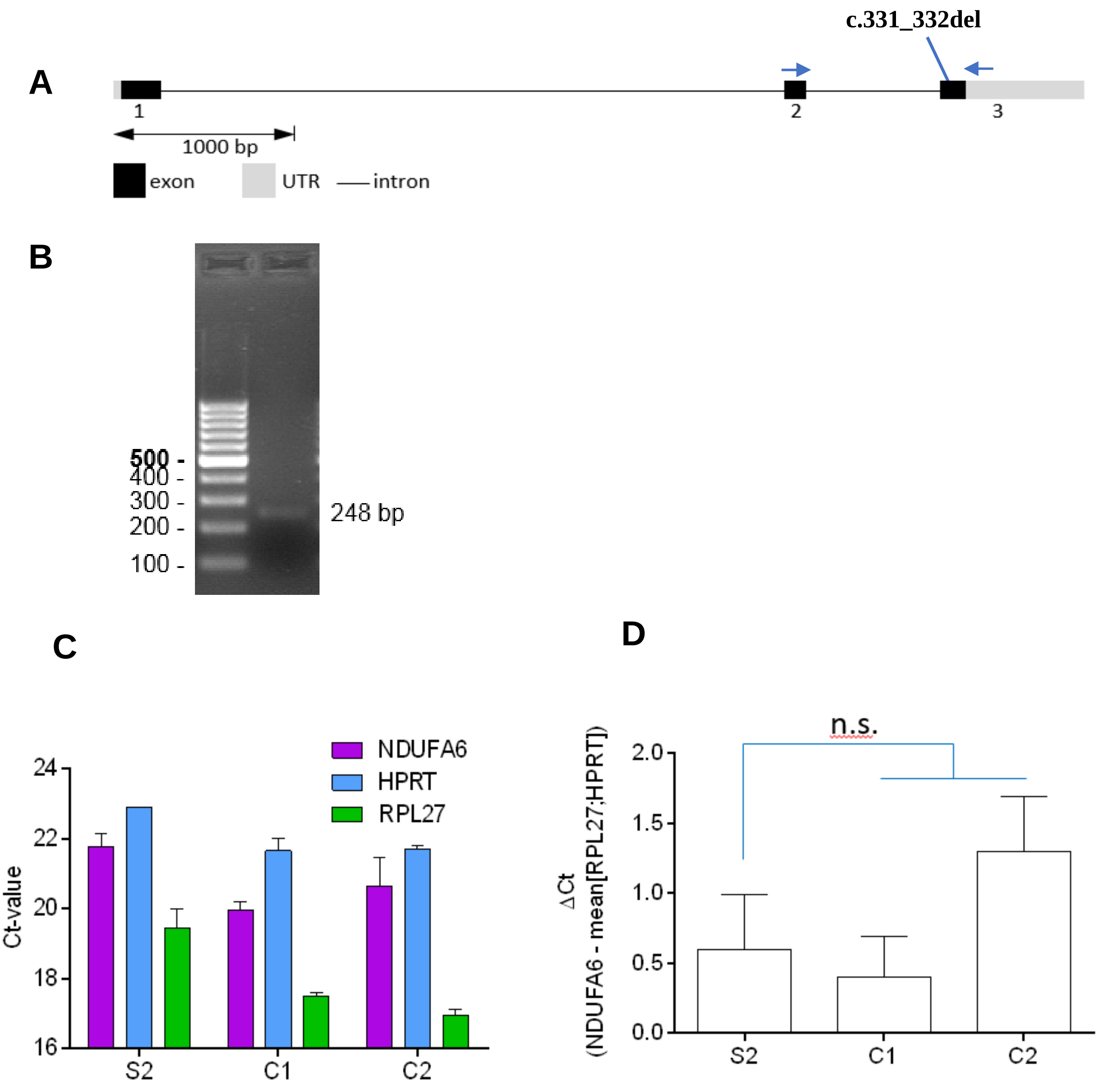

Figure S1. Normal amount of *NDUFA6* cDNA from fibroblasts in subject 2 (S2). A cDNA was prepared by reverse transcription using oligo-dT primers. Quantitative PCR of NDUFA6 was performed with a forward primer (5'-TGAAAATGGGACGGGATAAA-3') and reverse primer binding downstream of the frame-shift variant c.331_332del (5'-CAACGTGCATCTTTCCACTG-3'). Arrows indicate primer binding locations. **B** Agarose gel electrophoresis showed normal size (248 bp) of the *NDUFA6* PCR product. **C** Cycle threshold values for the NDUFA6 transcripts of S2 and two controls (C1, C2) are shown. **D** No difference in expression of NDUFA6 was seen between S2 and controls (using Student's unpaired T-test). Error Bars indicate the standard deviation, the experiments were performed in triplicates.

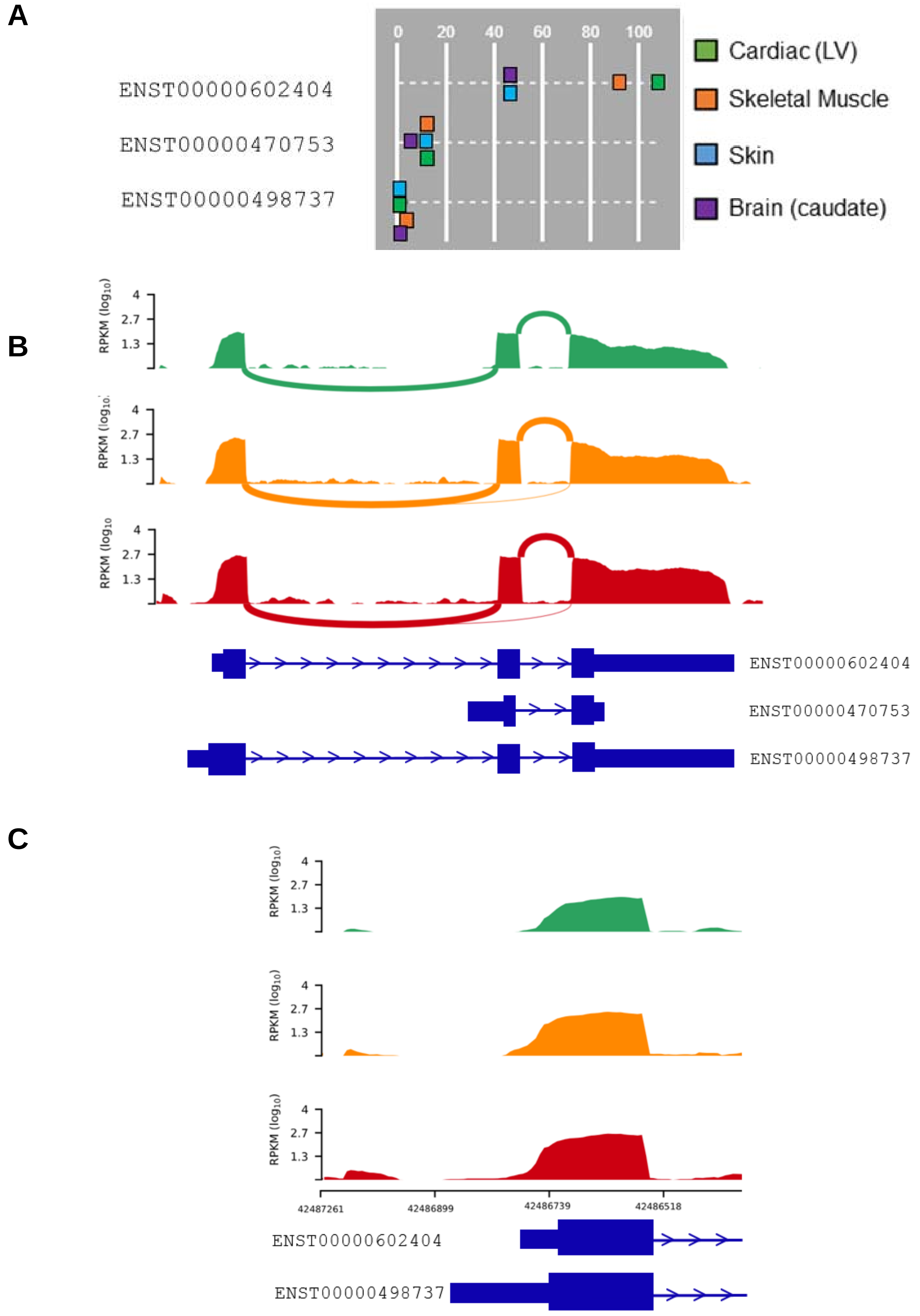

Figure S2. Alternative NDUFA6 transcripts and transcriptomics. A. Comparative RNAseq expression data obtained from the GTex repository supports highest expression of

the ENST00000602404 transcript in cardiac muscle, skeletal muscle, brain and skin (fibroblasts), with considerably lower expression of the alternative transcripts ENST00000498737 and ENST00000470753 (data correct at 04.04.2018). **B.** In-house RNAseq data supports the GTex data, providing further evidence that the ENST00000602404 transcript is predominantly expressed in skeletal muscle (green), cardiac muscle (red) and fibroblasts (orange). **C.** RNA expression at the 5'UTR corroborates ENST00000602404 as the predominant transcript in skeletal muscle (green), cardiac muscle (red) and fibroblasts (orange), with the transcription start site corresponding to that of ENST00000602404 and not ENST00000498737.

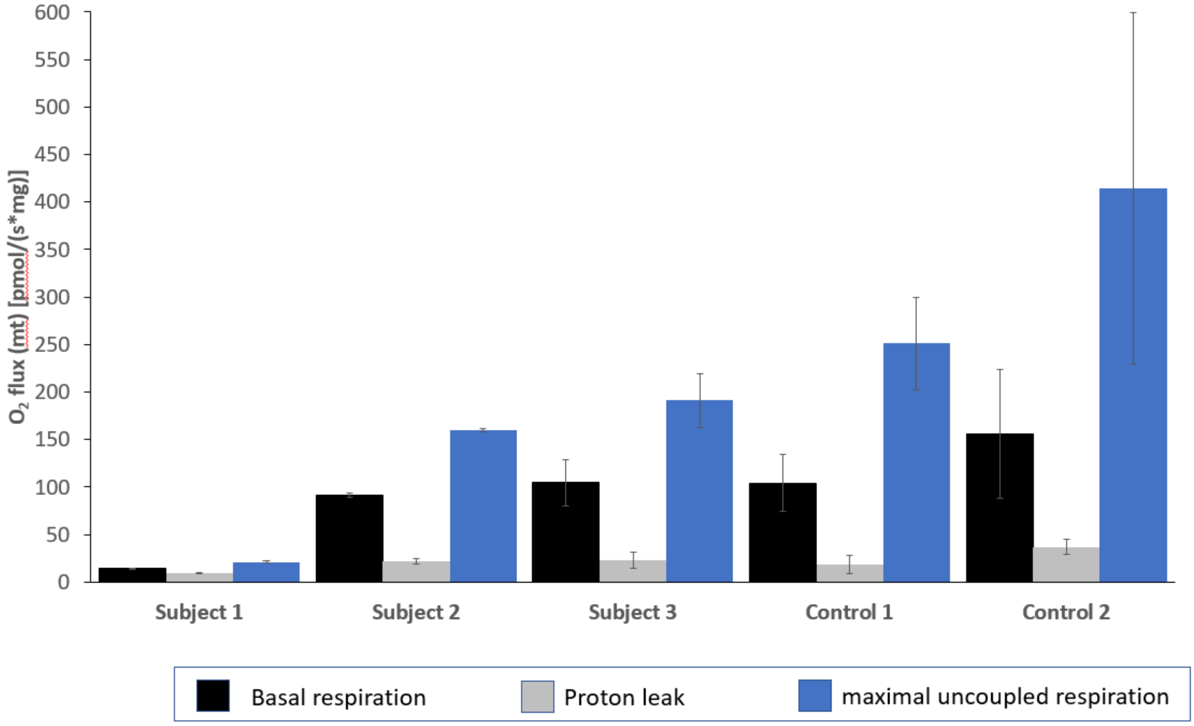

Figure S3. High resolution respirometry of Subject fibroblast cell lines.

Respiration of subject-derived fibroblasts compared to control fibroblasts shows basal respiration (**black**), oligomycin-inhibited (proton leak) respiration (**grey**) and maximum uncoupled respiration (**blue**) corrected for non-mitochondrial oxygen consumption. Data are mean ± SD from at least 2 experiments.

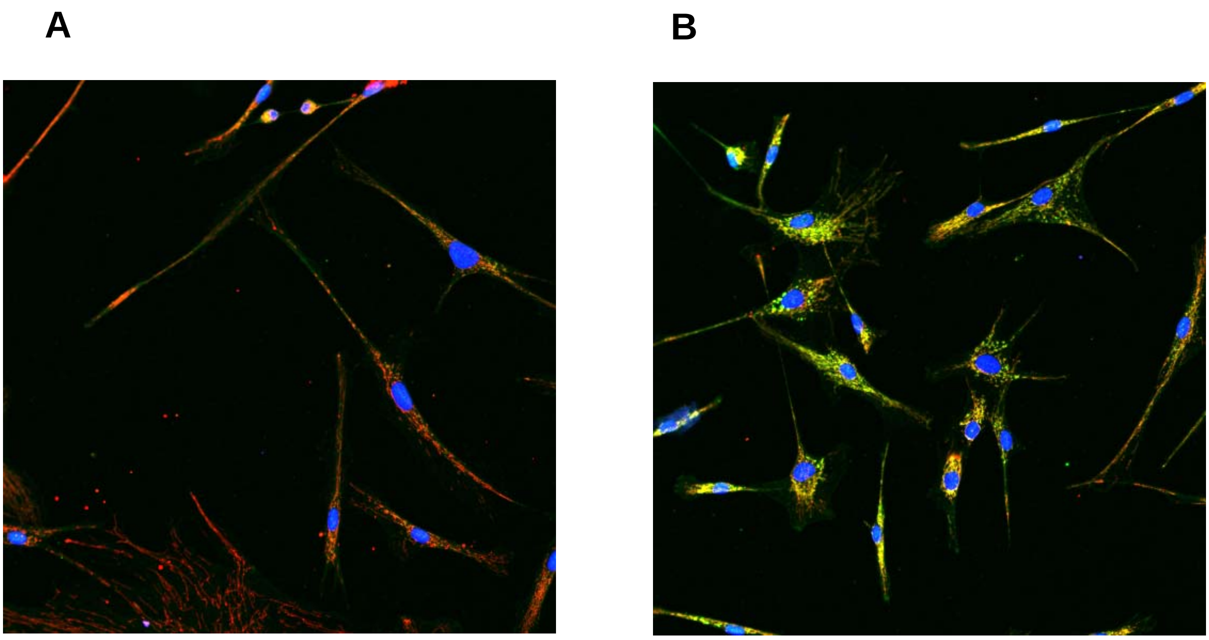

Figure S4: Cultured skin fibroblasts from individual S2 express a complex I defect
Immunofluorescence staining of fibroblasts obtained from individual S2 and a control was performed as reported[1] using the following primary antibodies: mouse monoclonal anti-NDUFS4 antibody detected in green (1:200; ab137064, Abcam, Cambridge, UK) and rabbit polyclonal anti-VDAC1 detected in red (1:400; ab15895, Abcam, Cambridge, UK) clearly demonstrating strong staining of the NDUFS4 (complex I) protein in the control (**B**) and absence in the patient cell line in the merged image (**A**). Scale bar = 50 µm.

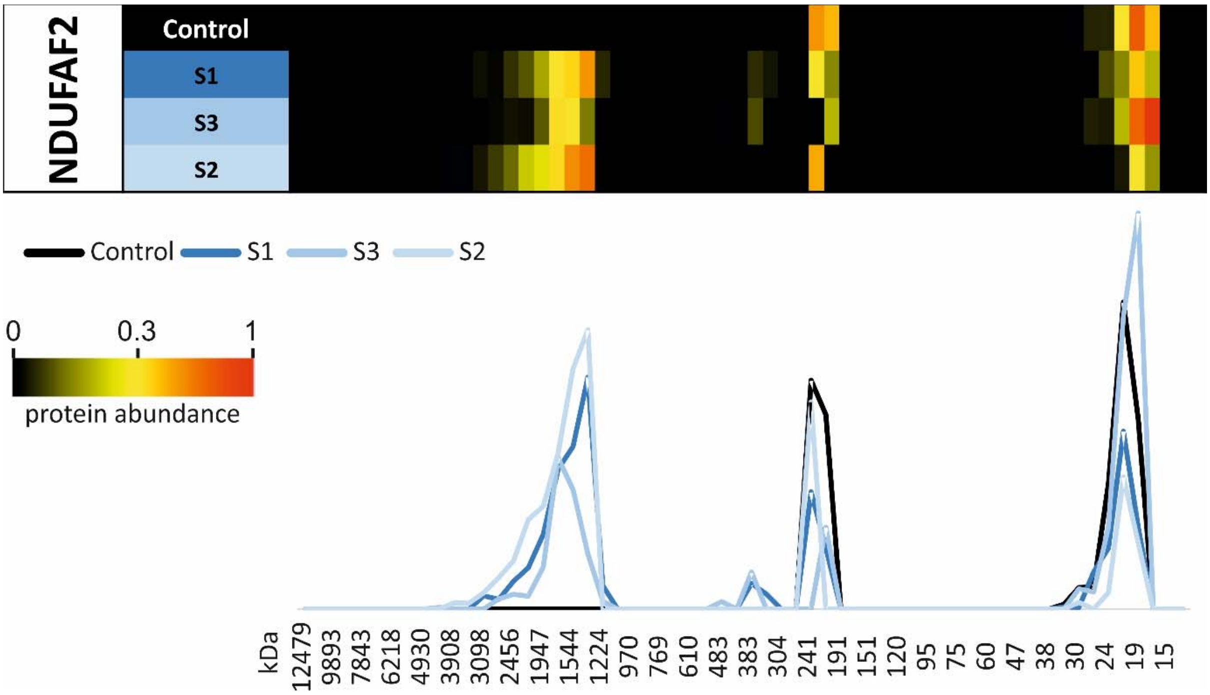

Figure S5. Retention of NDUFAF2 (mimitin) in Subjects' complexome profiles. Excerpt of complexome profiling data reveals NDUFAF2 is detectable at significant levels in the subcomplex state of supercomplexes in Subject cells. The molecular weight (approximately 1.6MDa) is consistent with the N-module lacking respirasome, CI/$CIII_2$/CIV. There is no evidence of NDUFAF2 in the supercomplex state of control fibroblast cell lines, suggesting that in subjects harbouring pathogenic *NDUFA6* variants, NDUFAF2 fails to dissociate from the assembly intermediate and enables supercomplex formation despite the absent N-module.

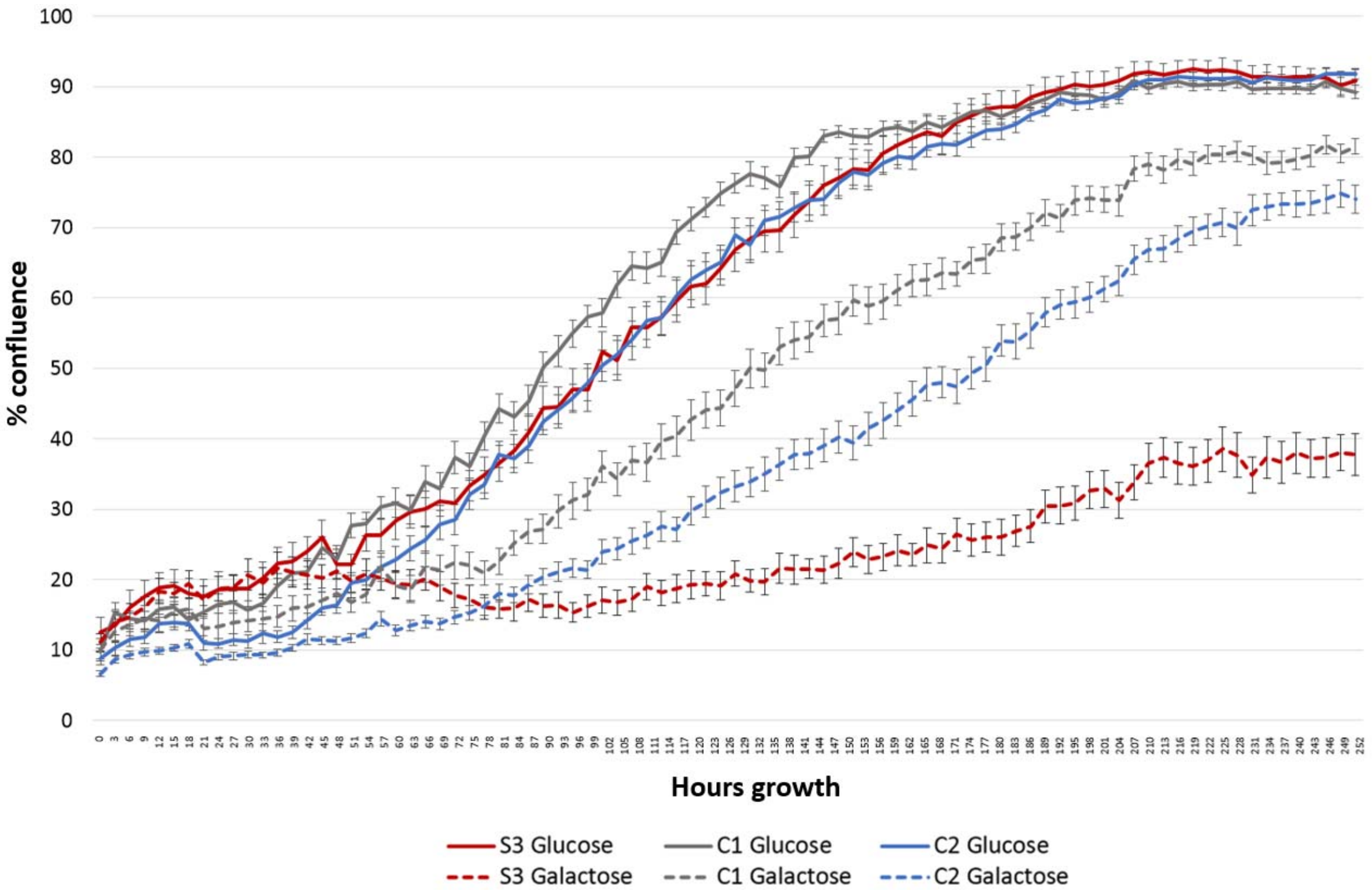

Figure S6. Glucose vs Galactose growth curves for fibroblasts from Subject 3.

Fibroblasts of Subject 3 and two controls were seeded, at 4,000 cells per well, into a 96 well plate (32 wells per cell line) in high glucose cell media containing 4.5 mg/ml glucose. After 15 hours, all media was removed and cells were washed with phosphate buffered saline. High glucose media was then returned to 16 wells per cell line, while the remaining 16 wells were switched to media containing 0.9 mg/ml galactose. Cell confluence was measured every 3 hours using the Incucyte® Live Cell Analysis System for 10 days. The fibroblasts of S3 demonstrated a delayed recovery of cell growth and significantly decreased growth rate in galactose media when compared with controls consistent with defective oxidative phosphorylation.

Supplemental Table 1. Details of commercially-available primary antibodies used in this study.

Primary antibody	Source	Catalogue number
NDUFV1	Proteintech	11238-1-AP
NDUFA13	Abcam	ab110240
MT-ND1	gift from Dr Anne Lombes	n/a
NDUFB8	Abcam	ab110242
NDUFA9	Abcam	ab14713
SDHA	Abcam	ab14715
VDAC1	Abcam	ab14734
UQCRC2	Abcam	ab14745
MT-COI	Abcam	ab14705
ATP5F1A	Abcam	ab14748

Supplemental references

1. Ahting, U., Mayr, J.A., Vanlander, A.V., Hardy, S.A., Santra, S., Makowski, C., Alston, C.L., Zimmermann, F.A., Abela, L., Plecko, B., *et al.* (2015). Clinical, biochemical, and genetic spectrum of seven patients with *NFU1* deficiency. Frontiers in genetics **6**, 123.
