# Supplementary material for: Bi-allelic Mutations in NDUFA6 Establish Its Role in Early-Onset Isolated Mitochondrial Complex I Deficiency
Source: Am J Hum Genet. 2018 Sep 20;103(4):592–601. doi: 10.1016/j.ajhg.2018.08.013 (PMC6174280; doi:10.1016/j.ajhg.2018.08.013)
Supplement: Document S2. Article plus Supplemental Data [file mmc2.pdf]

Bi-allelic Mutations in *NDUFA6* Establish Its Role in Early-Onset Isolated Mitochondrial Complex I Deficiency

Charlotte L. Alston,[1] Juliana Heidler,[2] Marris G. Dibley,[3] Laura S. Kremer,[4,5] Lucie S. Taylor,[1] Carl Fratter,[6] Courtney E. French,[7] Ruth I.C. Glasgow,[1] René G. Feichtinger,[8] Isabelle Delon,[9] Alistair T. Pagnamenta,[10] Helen Dolling,[7,11] Hugh Lemonde,[12] Neil Aiton,[13] Alf Bjørnstad,[14] Lisa Henneke,[15] Jutta Gärtner,[15] Holger Thiele,[16] Katerina Tauchmannova,[17] Gerardine Quaghebeur,[18] Josef Houstek,[17] Wolfgang Sperl,[8] F. Lucy Raymond,[7,9,11] Holger Prokisch,[4,5] Johannes A. Mayr,[8] Robert McFarland,[1] Joanna Poulton,[19] Michael T. Ryan,[3] Ilka Wittig,[2,20,21] Marco Henneke,[15,22] and Robert W. Taylor[1,22,*]

Isolated complex I deficiency is a common biochemical phenotype observed in pediatric mitochondrial disease and often arises as a consequence of pathogenic variants affecting one of the ~65 genes encoding the complex I structural subunits or assembly factors. Such genetic heterogeneity means that application of next-generation sequencing technologies to undiagnosed cohorts has been a catalyst for genetic diagnosis and gene-disease associations. We describe the clinical and molecular genetic investigations of four unrelated children who presented with neuroradiological findings and/or elevated lactate levels, highly suggestive of an underlying mitochondrial diagnosis. Next-generation sequencing identified bi-allelic variants in *NDUFA6*, encoding a 15 kDa LYR-motif-containing complex I subunit that forms part of the Q-module. Functional investigations using subjects' fibroblast cell lines demonstrated complex I assembly defects, which were characterized in detail by mass-spectrometry-based complexome profiling. This confirmed a marked reduction in incorporated NDUFA6 and a concomitant reduction in other Q-module subunits, including NDUFAB1, NDUFA7, and NDUFA12. Lentiviral transduction of subjects' fibroblasts showed normalization of complex I. These data also support supercomplex formation, whereby the ~830 kDa complex I intermediate (consisting of the P- and Q-modules) is in complex with assembled complex III and IV holoenzymes despite lacking the N-module. Interestingly, RNA-sequencing data provided evidence that the consensus RefSeq accession number does not correspond to the predominant transcript in clinically relevant tissues, prompting revision of the *NDUFA6* RefSeq transcript and highlighting not only the importance of thorough variant interpretation but also the assessment of appropriate transcripts for analysis.

Isolated complex I deficiency (OMIM: 252010) is a common biochemical phenotype observed in pediatric mitochondrial disease. The associated clinical and genetic heterogeneity is vast—individuals can present with a plethora of clinical symptoms ranging from isolated myopathy to Leigh syndrome (OMIM: 256000). Complex I is the first and largest complex of the mitochondrial respiratory chain and comprises 45 structural subunits with a minimal contingent of 20 ancillary proteins required for assembly and/or biogenesis.[1] The genes encoding these proteins involve either the mitochondria's own genetic material (mtDNA) or a nuclear-encoded gene of mitochondrial function,[2] and to date, causative defects have been identified in 39 genes encoding either complex I structural subunits or assembly factors.[3,4] Additionally, complex I deficiency can occur as a secondary consequence of dysfunction involving alternative mitochondrial processes.[5,6] Application of next-generation sequencing in the form of panel-based target capture or whole-exome sequencing has already demonstrated its utility in the

[1]Wellcome Centre for Mitochondrial Research, Institute of Neuroscience, Medical School, Newcastle University, Newcastle upon Tyne NE2 4HH, UK; [2]Functional Proteomics, SFB 815 Core Unit, Faculty of Medicine, Goethe-University, 60590 Frankfurt am Main, Germany; [3]Department of Biochemistry and Molecular Biology, Monash Biomedicine Discovery Institute, Monash University, 3800 Melbourne, Australia; [4]Institute of Human Genetics, Technische Universität München, 81675 Munich, Germany; [5]Institute of Human Genetics, Helmholtz Zentrum München, 85764 Neuherberg, Germany; [6]Oxford Medical Genetics Laboratories, Oxford University Hospitals NHS Foundation Trust, Churchill Hospital, Oxford OX3 7LE, UK; [7]Department of Medical Genetics, Cambridge Institute for Medical Research, University of Cambridge, Cambridge CB2 0XY, UK; [8]Department of Pediatrics, Salzburger Landeskliniken and Paracelsus Medical University, 5020 Salzburg, Austria; [9]Cambridge University Hospitals NHS Foundation Trust, Cambridge Biomedical Campus, Cambridge CB2 0QQ, UK; [10]National Institute for Health Research Oxford Biomedical Research Centre, Wellcome Centre for Human Genetics, University of Oxford, Oxford OX3 7BN, UK; [11]NIHR BioResource – Rare Diseases, Cambridge University Hospitals NHS Foundation Trust, Cambridge Biomedical Campus, Cambridge CB2 0QQ, UK; [12]Department of Inherited Metabolic Disease, Guy's and St. Thomas' NHS Foundation Trusts, Evelina London Children's Hospital, London SE1 7EH, UK; [13]Trevor Mann Baby Unit, Brighton and Sussex University Hospitals NHS Trust, Brighton BN2 5BE, UK; [14]Department of Pediatrics, Drammen Sykehus, 3004 Drammen, Norway; [15]Department of Pediatrics and Adolescent Medicine, Division of Pediatric Neurology, University Medical Center Göttingen, 37075 Göttingen, Germany; [16]Cologne Center for Genomics, University of Cologne, 50931 Cologne, Germany; [17]Institute of Physiology, Czech Academy of Sciences, 142 20 Prague, Czech Republic; [18]Department of Neuroradiology, Oxford University Hospitals NHS Foundation Trust, Oxford OX3 9DU, UK; [19]Nuffield Department of Women's and Reproductive Health, University of Oxford, Oxford OX3 9DU, UK; [20]German Center of Cardiovascular Research, Partner Site Rhein Main, 60590 Frankfurt am Main, Germany; [21]Cluster of Excellence "Macromolecular Complexes," Goethe-Universität, 60590 Frankfurt am Main, Germany
[22]These authors contributed equally to this work
*Correspondence: robert.taylor@ncl.ac.uk
https://doi.org/10.1016/j.ajhg.2018.08.013.
© 2018 The Authors. This is an open access article under the CC BY license (http://creativecommons.org/licenses/by/4.0/).

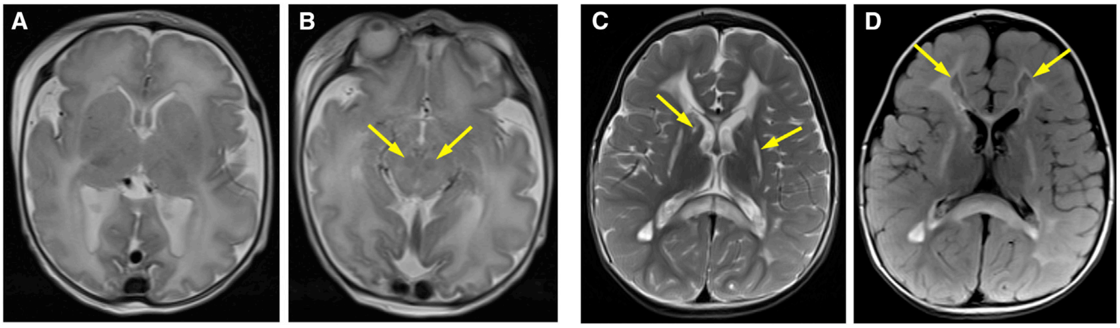

Figure 1. Neuroimaging of Subjects 1 and 2
(A and B) Axial T2 imaging of subject 1 shows diffusely abnormal hyperintensity of the entire white matter and decreased cortical folding for her age (A). Signal abnormalities within the brain stem and strikingly low signal affecting red nuclei (arrowheads) are also apparent (B).
(C) Axial T2 imaging of subject 2 shows extensive signal abnormalities of the cerebral white matter, including the corpus callosum. In addition, basal ganglia are affected, especially the putamen and caudate nucleus (arrowheads).
(D) Axial FLAIR imaging of subject 2 shows partial cystic degeneration of the affected cerebral white matter (arrowheads).

diagnosis of heterogeneous conditions such as mitochondrial disease and has been the catalyst for disease-associated gene discovery through its application to undiagnosed cohorts.[7] Facilitated by the GeneMatcher tool,[8] we report the findings from four unrelated, clinically affected children who presented with symptoms suggestive of mitochondrial disease.

Subject 1, a female infant, was the first child of healthy non-consanguineous Hungarian parents. On antenatal anomaly scan, she was noted to be small for her gestational age and have intracranial ventriculomegaly. Assessment by fetal medicine confirmed symmetrical growth restriction on the 3rd percentile, and fetal MRI at 30 weeks of gestation confirmed ventriculomegaly with mild cerebellar hypoplasia. She was born at term (37 + 3 weeks) by elective caesarean section for intrauterine growth restriction. Her birth weight was 1,660 g (<<0.4th percentile [<−4 SD]), and head circumference was 32.5 cm (2nd–9th percentile). At delivery, she required five inflation breaths and was initially placed on continuous positive airway pressure. Venous cord gas showed a pH of 7.25 and a base excess (BE) of −5.00. Within 40 min, she was self-ventilating in room air, but a capillary blood gas at that time revealed metabolic acidosis (pH = 7.12, lactate = 5.9 mmol/L, BE = −10.2) and hypoglycaemia (blood glucose = 1.2 mmol/L). A 10% dextrose infusion (60 mL/kg/day) was commenced, and benzylpenicillin and gentamicin were administered. Subsequent capillary blood gas analyses revealed an improvement in the metabolic acidosis but persistent hypoglycaemia (pH = 7.31, glucose = 1.8 mmol/L). A further bolus of 10% dextrose was administered before the infusion rate was increased to 90 mL/kg/day. Although the metabolic acidosis showed some improvement, the capillary blood lactate remained elevated (despite boluses of 10% dextrose and 0.9% saline). Repeat blood analyses revealed clotting abnormalities, hyperammonaemia (129 μM; normal < 100 μM), and a rising blood lactate (9.3 mmol/L; normal < 2.2 mmol/L). An abdominal ultrasound showed echogenic linear areas in the liver and normal kidneys. A second dose of vitamin K, followed by fresh-frozen plasma (FFP), sodium bicarbonate, 0.9% saline bolus, and frusemide, was administered. Apnoea at around 24 hr of age necessitated intubation and artificial ventilation before transfer to the regional neonatal intensive-care unit. Clinical examination demonstrated severe generalized hypotonia and absent primitive reflexes. Cerebral function monitoring (amplitude-integrated electroencephalography) showed a significantly suppressed baseline and no detectable seizure pattern. Cranial MRI undertaken at just over 36 hr of age demonstrated a diffusely abnormal signal intensity of the entire supratenterial white matter and decreased cortical folding for her age (Figure 1A), as well as of the brainstem (Figure 1B) and cerebellar white matter. Over the next 12 hr, despite extensive resuscitative efforts, including concomitant administration of three different inotropes (dopamine, dobutamine, and noradrenaline) and further FFP and packed red cell transfusion, the pupils became fixed and dilated, and she continued to deteriorate. Clotting remained abnormal (international normalized ratio = 3.2; normal < 1.1), although with the exception of gamma-glutamyl transpeptidase (1,225 IU; normal < 271 IU), liver-function tests were normal. Lactic acidosis exhibited relentless progression with a blood lactate reading in excess of 20 mM immediately before death at approximately 51 hr of age.

Subject 2 is the first son of healthy second-cousin Kurdish parents, who have a healthy younger daughter. He was born by normal vaginal delivery at 36 weeks of gestation (birth weight = 2.8 kg [50th percentile]) after an uneventful pregnancy. He spoke his first words at 12 months and at 16 months was walking independently. By 2 years, his parents noted a general physical weakness, and at 25 months, shortly after an unexplained fever, his gait deteriorated. Further motor regression and rapid onset of spasticity of all four limbs followed. He developed swallowing difficulties and required a gastrostomy tube for feeding. At 2.5 years, he was bedridden and was unable

to walk, grasp, or speak. He showed some recovery over subsequent months, during which he regained several words, and by 3 years was walking with assistance. Although there have been no further episodes of rapid regression, he has slowly deteriorated and has displayed dysarthric speech and wheelchair dependency from 7 years of age. Over the last 2 years, he has lost all voluntary movement in his limbs. Speech is just about understandable, and cognitive abilities remain good, but his mood is very low. He has an implanted pump for intrathecal Baclofen therapy and receives Botox injections every 3 months. Vison is impaired as a result of optic atrophy, whereas hearing is normal. Routine laboratory investigations revealed normal lactate levels in blood (1.2 mmol/L; normal < 2.2 mmol/L) and cerebrospinal fluid (CSF) (1.8 mmol/L; normal < 2.4 mmol/L). Light microscopy and routine histology of skeletal muscle biopsy did not exhibit any specific findings. Initial cranial MRI showed extensive and progressive disseminating lesions in supratentorial white-matter regions, including U-fibers and the corpus callosum, in the basal ganglia, brainstem, and cervical spinal cord, some of which enhanced with contrast (Figure 1C). Magnetic resonance spectroscopy revealed elevated lactate in multiple areas. On follow-up MRI, the brain abnormalities were less swollen, did not enhance, and displayed partial cystic degeneration and white-matter volume loss (Figure 1D).

Subject 3, a male infant, was born to first-cousin South Asian parents after a pregnancy complicated by oligohydramnios and intrauterine growth restriction. Delivery was by emergency caesarean section at 36 weeks, and although he did not require resuscitation at birth, he was noted to be small for his gestational age (2.27 kg [9th percentile]) and had minor dysmorphic features, including an accessory nipple, mild hypospadias, and sparse, fine scalp hair. At 3 weeks old, he became extremely irritable but continued to develop normally until 3 months, when he experienced an explosive onset of seizures. At 5 months old, after being admitted to the hospital for status epilepticus, it was clear that he had lost previously acquired developmental skills and was exhibiting truncal hypotonia with limb and facial dystonia. Investigation of his seizure disorder revealed elevated CSF lactate (5.0 mmol/L; normal < 2.4 mmol/L) and increased T2 signal in the basal ganglia on cranial MRI. Seizures remained problematic despite medical treatment, and he also suffered significant gastro-esophageal reflux with multiple episodes of aspiration pneumonia to the extent that by 2.5 years, he had developed bronchiectasis. Long-standing neutropenia, first noted at 2 years, was treated with granulocyte colony-stimulating factor. By 5 years, his hair had grown thicker and more diffusely on his scalp, although head growth was poor (0.4th percentile) and development remained markedly delayed in all domains. At his current age of 10 years, he sits independently but is unable to stand or walk. He is mute but responds positively to familiar voices. Vision is significantly impaired in relation to bilateral optic atrophy. Re-evaluation of his symptoms included repeat cranial MRI, which confirmed progressive changes involving the deep cortical gray matter of the basal ganglia as well as a more diffuse atrophic process. A second lumbar puncture showed persistently elevated CSF lactate (5.6 mmol/L; normal < 2.4 mmol/L), and analysis of CSF neurotransmitters revealed slightly low homovanillic acid of 319 IU (normal = 362–955 IU). Subject 3 has a healthy younger sister. Two male second-cousins (whose parents are also consanguineous) died at the ages of 5 and 1.5 years from epilepsy; both were said to also have abnormal, sparse hair, but no DNA samples were available for analysis.

Subject 4, a female infant, was the first child of non-consanguineous white British parents, whose pregnancy was uneventful. The baby was born at full term by spontaneous vaginal delivery, and the baby required no special care (birth weight = 2.78 kg [>2nd percentile], head circumference = 34 cm). At 7 weeks, she was admitted to the hospital after displaying a series of unusual movements suggestive of a seizure disorder. On admission, infection was excluded, but high blood gas lactate was noted. She gradually deteriorated as a result of status epilepticus, apnoea episodes, and persistent lactic acidosis. Despite intensive-care management in a tertiary care center, her lactic acidosis continued until her death at 13 weeks of age. Blood pH levels ranged from 7.308 to 7.076, blood gas lactate ranged from 16.9 to 18.5 mmol/L (normal = 0.6–1.4 mmol/L), and the CSF lactate level was recorded at 10.9 mmol/L (normal = 1.1–2.2 mmol/L). MRI shortly after presentation identified white-matter changes especially in the corticospinal tract, brain stem, and thalamus. All clinical indicators were consistent with a diagnosis of mitochondrial disease, prompting confirmatory genetic analysis.

Muscle and skin biopsy were taken from subjects 1–3 and referred to local centers for metabolic investigations; additionally, array comparative genomic hybridization was performed for subject 3 but revealed no abnormalities. Respiratory-chain analyses of muscle biopsy confirmed isolated complex I deficiency in subjects 1 and 2 (44% and 46% of control subjects, respectively), whereas analysis of muscle from subject 3 revealed normal complex I enzymology despite clinically suggestive features of mitochondrial disease. Diagnostic studies of respiratory-chain complex assembly (2D Blue Native polyacrylamide gel electrophoresis [BN-PAGE]) were undertaken in fibroblasts from subject 3 and revealed a marked complex I assembly defect in isolation, supporting a clinical diagnosis of complex I deficiency. Informed consent for diagnostic and research studies was obtained for all subjects in accordance with the Declaration of Helsinki protocols and approved by local institutional review boards.

Molecular genetic investigations were initiated for all four subjects—sequencing analysis of the entire mitochondrial genome (mtDNA) revealed only wild-type sequence, suggestive of an underlying Mendelian genetic

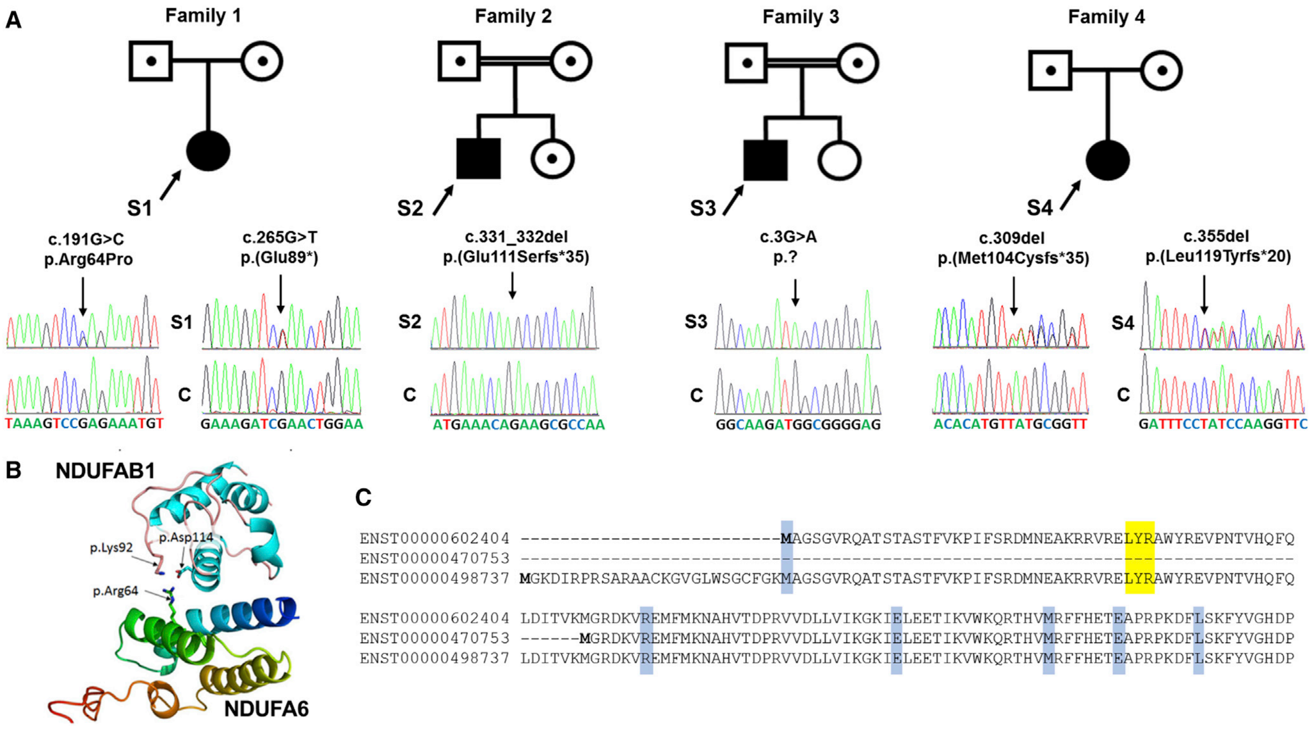

Figure 2. Bi-allelic *NDUFA6* Variants Are Identified in Four Unrelated Subjects

(A) Family pedigrees of subjects 1–4 and corresponding sequencing chromatograms presenting compound-heterozygous c.191G>C (p.Arg64Pro) and c.265G>T (p.Glu89*) *NDUFA6* variants in subject 1, the homozygous *NDUFA6* deletion c.331_332del (p.Glu111Serfs*35) in subject 2, the homozygous c.3G>A (p.?) *NDUFA6* variant in subject 3, and compound-heterozygous c.309del (p.Met104Cysfs*35) and c.355del (p.Leu119Tyrfs*20) *NDUFA6* frameshift mutations in subject 4. Abbreviations are as follows: S, subject; C, wild-type control.

(B) Analysis of the resolved crystal structure of complex I revealed spatial proximity of the Arg64 NDUFA6 residue with two negatively charged and highly conserved NDUFAB1 residues, Lys92 and Asp114. These most likely represent critical binding interactions between the two proteins during late complex I assembly.

(C) Alignment of the three in-frame *NDUFA6* transcripts demonstrates that ENST00000602404 (GenBank: NM_002490.5) uses an initiator methionine downstream of that used by ENST00000498737 (GenBank: NM_002490.4). The shortest transcript, ENST00000470753, initiates downstream of the highly conserved LYR motif (shaded yellow) that is reported to be critical for binding the mitochondrial acyl carrier (encoded by *NDUFAB1*); the function of this isoform therefore remains unknown. Subject variants are shaded blue.

etiology for each subject. Next-generation sequencing was undertaken in an attempt to elucidate their genetic diagnosis; a targeted approach was adopted for subject 1, as previously reported,[9] whereas unbiased whole-exome sequencing was performed for subjects 2 and 3. For subject 2, the exome library was prepared with the Illumina TruSeq Exome Enrichment Kit and sequenced on a HiSeq 1500; reads were aligned to the human genome (UCSC Genome Browser hg19). Bioinformatic analysis was performed as previously reported.[10] Genomic DNA from subject 3 and his parents was prepared and sequenced as a trio. Library capture and enrichment was performed with the NimbleGen SeqCap EZ Human Exome Library v.2.0 with subsequent sequencing on an Illumina HiSeq platform according to the manufacturer's protocols; data analysis was performed with Stampy v.1.0.20[11] and Platypus v.0.5.2.[12] For subject 4 and her parents, whole-genome sequencing and variant calling were performed as previously described.[13] Trio analysis was performed for *de novo*, homozygous, compound-heterozygous, and X-linked recessive inheritance patterns. In addition, mitochondrial genome variants were called and annotated with MToolBox.[14] Single-nucleotide variants, indels, structural variants, and copy-number variants were filtered for frequency (allele frequency < 1% in population datasets, including gnomAD and the ExAC Browser) and for functional impact (protein-coding changes or non-coding variants previously reported to be pathogenic in HGMD Professional or ClinVar). For each subject, variants were filtered to exclude those with a minor allele frequency (MAF) in excess of 1%, revealing likely causative variants in an accessory subunit of complex I, NDUFA6 (Figure 2A). Subject 1 harbored two heterozygous variants within *NDUFA6* (GenBank: NM_002490.5; OMIM: 602138), a c.191G>C transversion predicted to cause a p.Arg64Pro missense substitution and a c.265G>T transversion predicted to cause a premature stop codon, p.Glu89*. Subject 2 was found to harbor a homozygous 2 bp deletion, c.331_332del, predicted to cause a p.Glu111Serfs*35 frameshift, whereas subject 3 harbored a homozygous c.3G>A transition predicted to cause the abolition of the initiation methionine (p.?). Subject 4 was found to harbor two heterozygous frameshift

variants, c.309del (p.Met104Cysfs*35) and c.355del (p.Leu119Tyrfs*20), *in trans*. Analysis of parental DNA samples supported recessive inheritance of each subject's *NDUFA6* variants; the unaffected sister of subject 2 was heterozygous for the familial c.331_332del (p.Glu111Serfs*35) *NDUFA6* variant. The c.265G>T (p.Glu89*) variant (rs758833609) is reported in two heterozygous individuals in gnomAD (MAF = 0.0008%). The c.3G>A (p.?) variant (rs1023075742) is reported in one heterozygous individual in gnomAD (MAF = 0.0004%), as is the c.355del (p.Leu119Tyrfs*20) variant (rs781099275). The c.309del (p.Met104Cysfs*35) variant (rs763006208) is reported in 16 heterozygous individuals in gnomAD (MAF = 0.006%). The c.191G>C (p.Arg64Pro) and c.331_332del (p.Glu111Serfs*35) variants are unreported in gnomAD, ClinVar, and dbSNP. None of the subjects' *NDUFA6* variants are reported in the homozygous state in gnomAD, consistent with a very rare status and offering preliminary support for a deleterious etiology. All *NDUFA6* variants have been submitted to ClinVar (see Accession Numbers).

The c.3G>A (p.?), c.265G>T (p.Glu89*), c.309del (p.Met104Cysfs*35), c.331_332del (p.Glu111Serfs*35), and c.355del (p.Leu119Tyrfs*20) *NDUFA6* variants can be classified as pathogenic according to American College of Medical Genetics and Genomics guidelines,[15] whereas the c.191G>C (p.Arg64Pro) variant is predicted to be likely pathogenic. The c.191G>C (p.Arg64Pro) variant affects 1 of 12 evolutionarily conserved arginine residues, and *in silico* predictions are strongly supportive of pathogenicity (Align-GVGD = C65, Grantham distance = 102.71, SIFT = 0.000 [deleterious], PolyPhen = 1.000 [probably damaging], raw CADD = 7.35, scaled CADD = 34). CADD scores are often regarded as the most accurate predictor of pathogenicity, and a scaled CADD score of 34 is strongly supportive of a deleterious consequence.[16] Investigation of the Protein Databank model of NDUFA6 (PDB: 5XTD.E) confirms that the proximity of the Arg64 residue to critical residues of NDUFAB1 could inhibit binding of NDUFAB1 when mutated (Figure 2B). The replacement of a charged and bulky arginine residue with a small, uncharged proline residue is unlikely to be tolerated, and because of its structural conformation, a proline residue is likely to create a kink in the alpha helix.[17] The c.331_332del variant harbored by subject 2 occurs within the terminal exon of the gene and is therefore predicted to evade nonsense-mediated mRNA decay.[18] In fact, investigation of cDNA from fibroblasts showed a normal amount of *NDUFA6* transcript (Figure S1). The encoded polypeptide is predicted to harbor 34 alternative amino acids at the C terminus, extending the polypeptide by 17 residues and affecting the tertiary structure of the mature protein and thus possibly resulting in proteolytic degradation.

It is noteworthy that NDUFA6 has three reported functional isoforms in UniProt: the first (UniProt: P56556) represents the longest isoform with 154 residues (GenBank: NM_002490.4; Ensembl: ENST00000498737.6), the second (UniProt: A0A0C4DGSO) has 128 residues (Ensembl: ENST00000602404.5), and the third (UniProt: R4GN43) has 71 residues (Ensembl: ENST00000470753.1). All isoforms occur in frame and use alternative methionine residues for translation initiation (Figure 2C). Until now, no consensus on the clinically relevant transcripts has been reached, but Genotype-Tissue Expression (GTEx) data and in-house RNA-seq data[19] support ENST00000602404.5 as the predominant isoform, and RNA expression data specifically correlate with the 5′ UTR of ENST00000602404.5 (Figure S2). These data have led ENST00000602404.5 to now be associated with the revised accession number GenBank: NM_002490.5.

In order to further characterize the functional consequence of the subjects' *NDUFA6* variants, we undertook functional investigations with fibroblast cell lines from each subject for whom tissues were available (S1–S3). High-resolution respirometry of subject fibroblasts was performed with the Oroboros 2k platform as described previously[20] and revealed a remarkable impairment of intact cell respiration in subject 1. Intact cell respiration of subjects 2 and 3 was almost comparable to that of control fibroblasts (Figure S3). BN-PAGE of enriched mitochondrial fractions from subject fibroblasts and age-matched control cell lines was performed as previously reported.[21] Immunoblotting using antibodies conjugated against structural subunits from each OXPHOS complex (complex I [NDUFB8], complex II [SDHA], complex III [UQCRC2, core 2], complex IV [MT-CO1], and complex V [ATP5F1A]) revealed a reduction of fully assembled complex I levels, whereas all other OXPHOS complexes were unaffected. Complex I subcomplexes, consistent with stalled assembly intermediates, were apparent in the subjects' cell lines, but not in control cells (Figure 3A). Subject 1, who presented with the most aggressive clinical course, had the most marked reduction in fully assembled complex I, whereas subjects 2 and 3 (whose presentations were less severe) also had impaired complex I assembly, although to a lesser extent. Steady-state levels of complex I structural components were assessed with SDS-PAGE of subject and control fibroblast cell lysates according to previous methodologies.[21] Immunoblotting using antibodies conjugated against various structural subunits of complex I (NDUFV1, NDUFA13, MT-ND1, NDUFA9 and NDUFB8) revealed a marked reduction in the steady-state levels of complex I subunits for subject 1, whereby the N module and Q module subunits were most affected (Figure 3B). A similar but milder pattern was observed for subjects 2 and 3, consistent with BN-PAGE analyses (Figure 3B). SDH70 (SDHA of complex II) and porin were used as markers. Immunofluorescence staining of fibroblasts with NDUFS4 as a marker of complex I expression revealed lower expression of complex I protein in subject 2 than in age-matched controls subjects (Figure S4). Given that pathogenic variants have not previously been reported in NDUFA6, fibroblast cell lines from subjects 1–3 were subject to lentiviral rescue as previously described.[24] Subsequent analysis by BN-PAGE

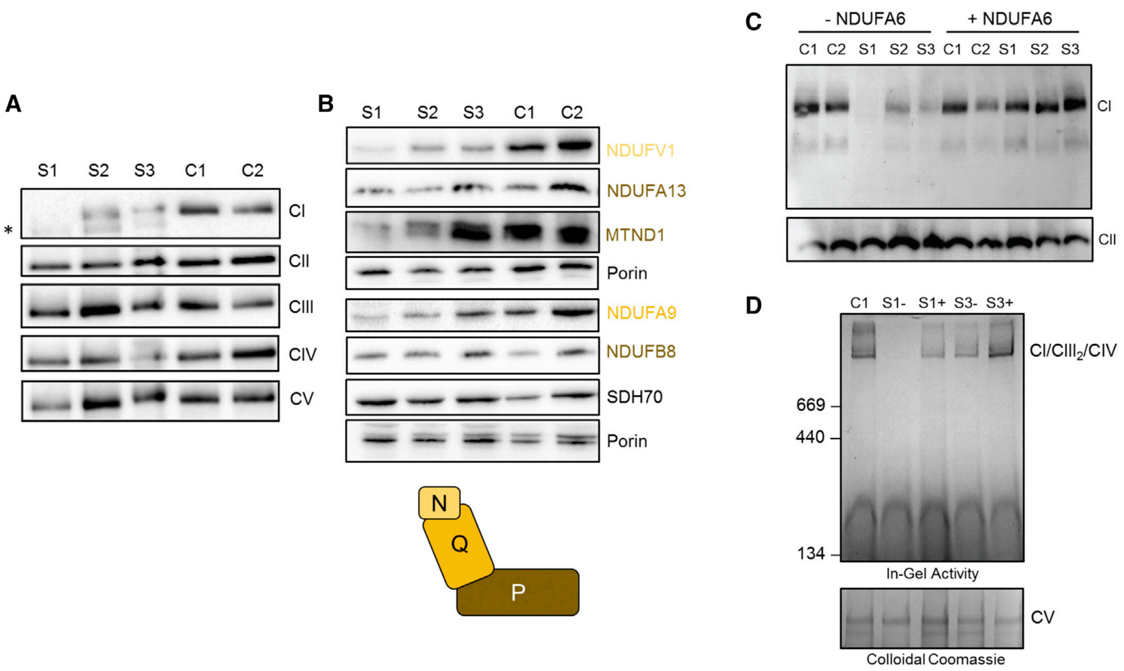

Figure 3. BN-PAGE, SDS-PAGE, and Complementation Studies

(A) Mitochondria isolated from cultured skin fibroblasts from subjects 1–3 and age-matched control subjects were solubilized in n-dodecyl β-D-maltoside (DDM) and subjected to BN-PAGE and immunoblotting analysis using antibodies directed to various OXPHOS complexes as indicated. The blot probed with an antibody raised against NDUFB8 revealed the presence of additional, partially assembled complex I intermediates in the samples from subjects 1–3 (indicated by an asterisk) but not in control samples.
(B) Whole-fibroblast cell lysates from subjects 1–3 and age-matched control subjects were analyzed by SDS-PAGE. Immunoblotting was performed with antibodies against complex I subunits or control proteins (SDHA and porin) as indicated. Complex I structural subunits are color coded according to their corresponding complex I modules, as illustrated in the complex I pictogram.
(C) Wild-type *NDUFA6* cDNA was generated and introduced into control and subject cell lines via retroviral expression. Whole-cell lysates were solubilized in 1% Triton X-100 (immunoblotting) before BN-PAGE analysis. Immunoblotting using antibodies against the complex I subunit NDUFA9 (top) and the complex II subunit SDHA (bottom) as a loading control revealed less complex I in subject cell lines than in control cell lines. After transfection with *NDUFA6*, complex I levels were restored.
(D) In-gel activity analysis was performed according to Zerbetto et al.[22] with mitochondria isolated from the fibroblasts of subjects 1 (S1) and 3 (S3) with (+) and without (−) lentiviral transduction with NDUFA6 cDNA and an aged-matched control subject; enriched mitochondria were solubilized in 1% digitonin for BN-PAGE analysis, which demonstrated restoration of complex I activity in subjects 1 and 3 after the introduction of *NDUFA6* (top). The gel was then stained with colloidal Coomassie according to Neuhoff et al.[23] as a loading control (bottom). All antibodies used are documented in Table S1.

confirmed that transduction of all three subject cell lines with a wild-type *NDUFA6* cDNA transcript ameliorated the complex I assembly defect, unequivocally establishing the subjects' *NDUFA6* variants as the cause of disease (Figure 3C). In-gel activity assay of enriched mitochondria derived from the fibroblast cell lines from subjects 1 and 3 recapitulated the phenotypic rescue with restoration of complex I activity (Figure 3D); analysis of the fibroblast cell line from subject 2 was attempted but was unsuccessful because cells failed to grow.

To further delineate the consequence of the subjects' *NDUFA6* variants on mitochondrial complex I assembly, we undertook complexome profiling of age-matched control cells (Figure 4A) and subject fibroblasts (Figure 4B) as previously described.[26,27] Analysis of complexome profiling data recapitulated the assembly defect observed after BN-PAGE analysis of fibroblasts from subjects 1–3 (Figures 4B and 4C). A clear reduction of N-module subunits was visible, particularly in the most severely affected individual, subject 1 (Figure 4B, boxed area), correlating the assembly defect with the observed phenotypic severity. A loss of NDUFA7 and NDUFA12 from the subcomplex is evident, particularly in subject 1, and appears to correlate with the degree of NDUFA6 loss. It is possible that NDUFA6 is a prerequisite for NDUFA7 and NDUFA12 incorporation into the complex I holoenzyme and that absence of NDUFA6 or its impaired function results in stalling and prevents subsequent interactions with the subunits of the N-module. Loss (subject 1; Figure 4B) or marked reduction (subjects 2 and 3; Figures 4C and 4D) of the mitochondrial acyl carrier (NDUFAB1) from complex I is also evident from complexome profiling data; this is unsurprising given that the LYR motif of NDUFA6 has been demonstrated to be critical for NDUFAB1 binding in the model aerobic yeast, *Yarrowia lipolytica*.[28] Interestingly, the complexome profiling data provide evidence of NDUFAF2, a late-stage complex I assembly factor, still bound to the stalled assembly intermediate (Figure S5); this has previously been observed as a consequence of pathogenic mutations affecting N-module subunits *NDUFV1* (OMIM: 161015) and *NDUFS4*

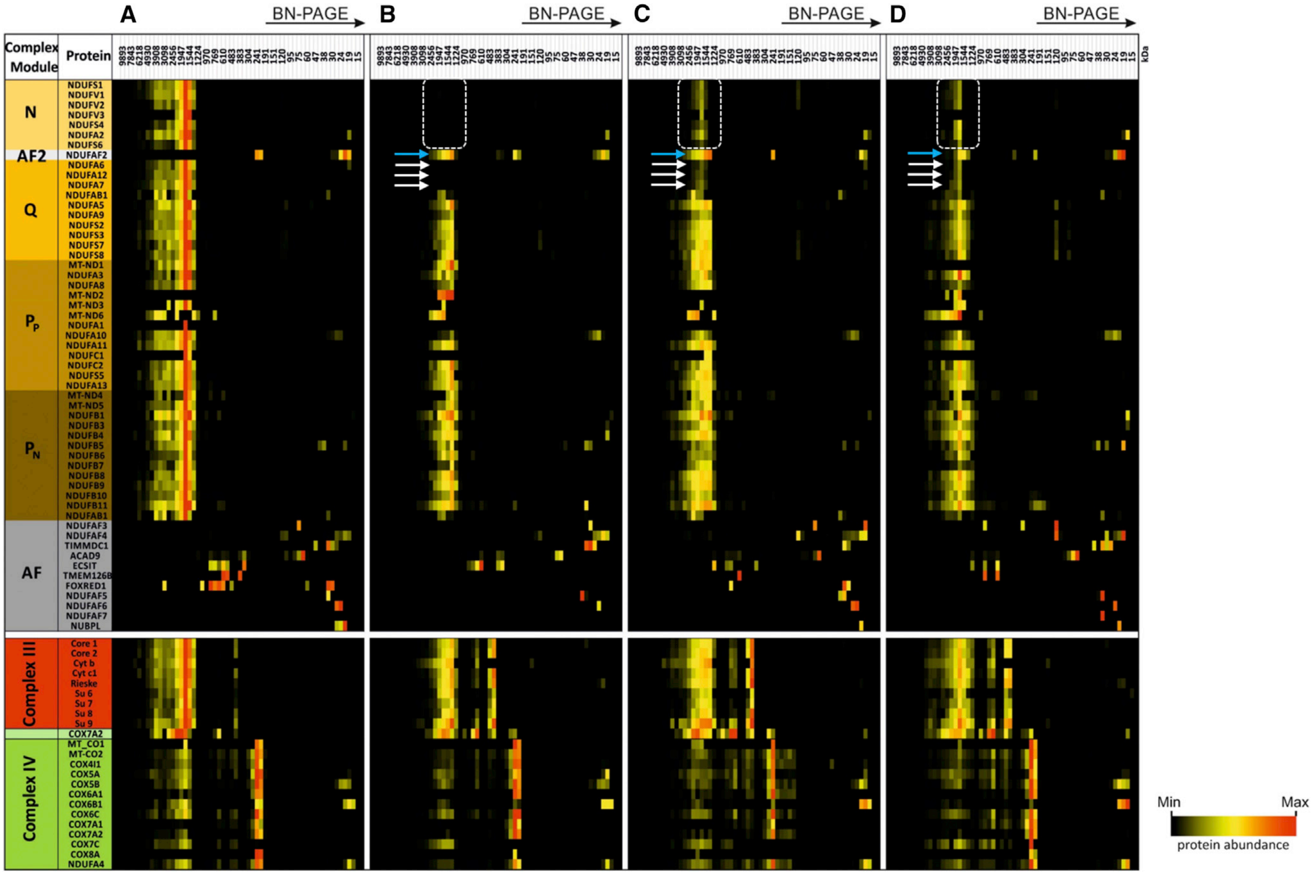

Figure 4. Complexome Profiling of Fibroblasts from Subjects 1–3 Identifies Stalled Complex I Assembly Intermediates
Control (A), subject 1 (B), subject 2 (C), and subject 3 (D). The left lane indicates assembly factors (gray) and structural subunits (orange-brown) of complex I modules, complex III (red), and complex IV (green). Dashed boxes indicate the loss or reduction of N-module subunits in subject fibroblast lines. White arrows highlight the loss or reduction of NDUFA6, NDUFA7 and NDUFA12 from complex I in subjects 1–3, and blue arrows indicate the location of NDUFAF2, still bound to the stalled complex I intermediate in subject complexomes but not in the control. Assignment of complex I subunits to modules was according to Wirth et al.[25] Intensity-based absolute quantification (iBAQ) values were normalized to the sum of all values of the control. Native mass calibration was performed according to Fuhrmann et al.[26]

(OMIM: 602694).[29] The failure of NDUFAF2 to dissociate from the stalled assembly intermediate (~830 kDa) after mutation of either N-module or Q-module subunits supports the theory that dissociation of NDUFAF2 represents a key checkpoint in complex I holoenzyme assembly.[30] Despite the absence of N-module subunits, subject complexome data support an ability of the 830 kDa intermediate (plus bound NDUFAF2) to participate in supercomplex formation, whereby fully assembled complexes III and IV appear in complex with the P- and Q-modules of complex I (minus NDUFA6, NDUFA7, NDUFA12, and the Q-module associated copy of NDUFAB1). This supercomplex ($I^{830}/III_2/IV_{1\text{-}4}$) does not represent a functional respirasome given its apparent lack of NADH dehydrogenase activity but is consistent with other findings showing that N-module subunits are not needed to act as scaffolding during supercomplex assembly.[31,32] This is also supported by the fact that not a single subunit of the N-module interacts with any of the structural subunits of either complex III or IV in the resolved native supercomplexes.[33]

Recently identified disease-related genes associated with a biochemical manifestation of complex I deficiency include the assembly factors *TIMMDC1* (OMIM: 615534)[19] and *TMEM126B* (OMIM: 615533),[34,35] the glutathione transporter *SLC25A10* (OMIM: 606794),[36] and *EXOSC3* (OMIM: 606489), a subunit of the human RNA exosome complex.[37] The connection between an apparent isolated complex I deficiency and the putative genetic defect is often not clear cut and can in fact occur as a secondary consequence of mitochondrial dysfunction in another pathway. For example, bi-allelic pathogenic variants in *MTFMT* (OMIM: 611766), encoding a mitochondrial methyltransferase that formylates methionine residues for use in mitochondrial protein initiation, often results in an isolated complex I deficiency despite affecting multiple OXPHOS complexes.[38] The isolated complex I deficiency observed in fibroblasts from subjects 1 and 2 is consistent with the bi-allelic *NDUFA6* variants identified given that *NDUFA6* encodes an accessory structural subunit of complex I. Importantly, NDUFA6 is one of 27 accessory subunits

that have been shown to be essential for complex I assembly and activity.[32] Subject 3, who lacks biochemical evidence of complex I dysfunction, remains somewhat enigmatic, particularly given that his homozygous *NDUFA6* variant is predicted to abolish the initiation methionine. It is common for subject fibroblasts to demonstrate normal complex I activities despite a marked deficiency in a clinically relevant tissue (e.g., skeletal muscle),[39] but the situation for subject 3—normal enzymatic activity in muscle despite a demonstrable complex I assembly defect—is most unusual. Although the fibroblast cell line from subject 3 also demonstrated normal complex I activity (data not shown), a clear growth defect was demonstrated when glucose-rich culture medium was replaced with galactose-rich (and glucose-free) medium, forcing the cells to be dependent upon oxidative phosphorylation for ATP production (Figure S6). The existence of the longer NDUFA6 isoform (UniProt: P56556) could explain the normal complex I activity reported for subject 3, whose variant would instead act as a homozygous missense variant in the encoding transcript (c.81G>A [p.Met27Ile]). *In silico* predictions suggest that the c.81G>A (p.Met27Ile) variant might be tolerated (Align-GVGD = C0, Grantham distance = 10.12, SIFT = 0.070 [tolerated], PolyPhen = 0.702 [possibly damaging], CADD = 34). The CADD score discordance is most likely due to the consideration of alternative transcripts in the prediction and is therefore exacerbated by the pathogenicity associated with the initiation methionine in the alternative transcript. Attempts to assess whether an alternative methionine was selected for translation initiation *in vivo* have been unsuccessful; analysis of tryptic fragments of subject proteins produced during complexome profiling was uninformative, and commercially available antibodies have failed to conjugate successfully with the target protein in subject and control tissues (data not shown). Another possibility is that NDUFA6 translation is subject to "leaky" initiation and thus results in selection of various in-frame methionine residues for translation initiation; this would result in the co-existence of Met1- and Met27-containing NDUFA6 polypeptides in the human mitoproteome and, in the context of subject 3, would dilute the pathogenic etiology. The additional isoform (P56556) is not anticipated to ameliorate the pathogenicity of the *NDUFA6* variants harbored by subject 1 or 2—their variants are predicted to exert the same effect in an isoform-independent manner.

This report describes bi-allelic *NDUFA6* variants in four pediatric subjects who each have a clinical diagnosis of mitochondrial disease. For the three subjects with available tissues, associated complex I assembly defects were demonstrated together with lentiviral rescue of the biochemical phenotype. This study therefore establishes NDUFA6 as the 29th structural subunit of complex I to be associated with human pathology. Given that one subject had normal complex I activity in skeletal muscle, this report epitomizes the importance of an unbiased approach to genetic diagnosis. Thorough variant interpretation and assessment of appropriate transcripts remain both an obligation and a challenge for the diagnostic community in this genomic era.

Accession Numbers

The accession numbers for variants c.3G>A, c.191G>C, c.265G>T, c.309del, c.331_332del and c.355del are ClinVar: SCV000787623, SCV000787624, SCV000787625, SCV000787626, SCV000787627, and SCV000787628, respectively.

Supplemental Data

Supplemental Data include six figures and one table and can be found with this article online at https://doi.org/10.1016/j.ajhg.2018.08.013.

Acknowledgments

Work in our laboratories is supported by the Wellcome Centre for Mitochondrial Research (203105/Z/16/Z), the MRC Centre for Neuromuscular Diseases (G0601943), the NIHR Biomedical Research Centre in Age and Age Related Diseases award to the Newcastle upon Tyne Hospitals NHS Foundation, the Newcastle Molecular Pathology Node, the NHS Highly Specialised Service for Rare Mitochondrial Disorders, the Lily Foundation, the Georg August University Göttingen Faculty of Medicine research program (to M.H.), the Deutsche Forschungsgemeinschaft (SFB 815, project Z1 to I.W. and GA354/14-1 to J.G.), the Centre of Mitochondrial Biology and Pathology (Czech Science Foundation grant 14-36804G), the German Bundesministerium für Bildung und Forschung through the German Network for Mitochondrial Disorders (mitoNET, 01GM1113), the E-Rare project GENOMIT (01GM1603), the EU Horizon2020 Collaborative Research Project SOUND (633974), the Wellcome Trust (090532/Z/09/Z), and the NIHR Biomedical Research Centre Oxford. C.L.A. received a NIHR doctoral fellowship (NIHR-HCS-D12-03-04). J.P. is supported by the MRC (MR/J010448/1) and the Wellcome Trust (0948685/Z/10/Z). M.T.R. is supported by Australian National Health and Medical Research Council grant 1125390. M.G.D. is supported by the Australian Mitochondrial Disease Foundation. J.A.M. is supported by the E-Rare project GENOMIT (I 2741-B26) and Austrian Science Fonds. F.L.R., C.F., and H.D. are supported by the Rosetrees Trust, Cambridge Biomedical Research Centre, and NIHR BioResource (RG65966). We thank Jana Meisterknecht for technical assistance, Christian Mertes for helping to create Figure S2, and Anne Lombes for the MTND1 antibody. The views expressed are those of the authors and not necessarily of the NHS, NIHR, or Department of Health.

Declaration of Interests

The authors declare no competing interests.

Received: May 1, 2018
Accepted: August 22, 2018
Published: September 20, 2018

Web Resources

AlignGVGD, http://agvgd.hci.utah.edu/agvgd_input.php
CADD, http://cadd.gs.washington.edu/
ClinVar, https://www.ncbi.nlm.nih.gov/clinvar/

Ensembl, www.ensembl.org/
ExAC Browser, http://exac.broadinstitute.org/
GenBank, http://www.ncbi.nlm.nih.gov/genbank/
GeneMatcher, https://genematcher.org/
gnomAD, http://gnomad.broadinstitute.org/
GTEx Portal, https://www.gtexportal.org/home/
OMIM, http://www.omim.org/
PolyPhen-2, http://genetics.bwh.harvard.edu/pph2/
RSCB Protein Data Bank, https://www.rcsb.org/pdb/home/home.do
SIFT http://sift.bii.a-star.edu.sg/
UCSC Genome Browser, https://genome.ucsc.edu/
UniProt, www.uniprot.org/

References

1. Formosa, L.E., Dibley, M.G., Stroud, D.A., and Ryan, M.T. (2018). Building a complex complex: Assembly of mitochondrial respiratory chain complex I. Semin. Cell Dev. Biol. *76*, 154–162.
2. Calvo, S.E., Clauser, K.R., and Mootha, V.K. (2016). MitoCarta2.0: An updated inventory of mammalian mitochondrial proteins. Nucleic Acids Res. *44* (D1), D1251–D1257.
3. Frazier, A.E., Thorburn, D.R., and Compton, A.G. (2017). Mitochondrial energy generation disorders: Genes, mechanisms and clues to pathology. J. Biol. Chem. Published online December 12, 2017. https://doi.org/10.1074/jbc.R117.809194.
4. Piekutowska-Abramczuk, D., Assouline, Z., Mataković, L., Feichtinger, R.G., Koňaříková, E., Jurkiewicz, E., Stawiński, P., Gusic, M., Koller, A., Pollak, A., et al. (2018). *NDUFB8* mutations cause mitochondrial complex I deficiency in individuals with Leigh-like encephalomyopathy. Am. J. Hum. Genet. *102*, 460–467.
5. Haack, T.B., Kopajtich, R., Freisinger, P., Wieland, T., Rorbach, J., Nicholls, T.J., Baruffini, E., Walther, A., Danhauser, K., Zimmermann, F.A., et al. (2013). *ELAC2* mutations cause a mitochondrial RNA processing defect associated with hypertrophic cardiomyopathy. Am. J. Hum. Genet. *93*, 211–223.
6. Freisinger, P., Haack, T., Kopajtich, R., Johannes, M., Ahting, U., Sperl, W., Plecko, B., Wilichowski, E., Meitinger, T., and Prokisch, H. (2013). Mitochondriopathy due to mutations in MTFMT: A predominant neurologic phenotype. Neuropediatrics *44*, FV12_03.
7. Ohtake, A., Murayama, K., Mori, M., Harashima, H., Yamazaki, T., Tamaru, S., Yamashita, Y., Kishita, Y., Nakachi, Y., Kohda, M., et al. (2014). Diagnosis and molecular basis of mitochondrial respiratory chain disorders: Exome sequencing for disease gene identification. Biochim. Biophys. Acta *1840*, 1355–1359.
8. Sobreira, N., Schiettecatte, F., Valle, D., and Hamosh, A. (2015). GeneMatcher: A matching tool for connecting investigators with an interest in the same gene. Hum. Mutat. *36*, 928–930.
9. Alston, C.L., Howard, C., Oláhová, M., Hardy, S.A., He, L., Murray, P.G., O'Sullivan, S., Doherty, G., Shield, J.P., Hargreaves, I.P., et al. (2016). A recurrent mitochondrial p.Trp22Arg *NDUFB3* variant causes a distinctive facial appearance, short stature and a mild biochemical and clinical phenotype. J. Med. Genet. *53*, 634–641.
10. Ploski, R., Pollak, A., Müller, S., Franaszczyk, M., Michalak, E., Kosinska, J., Stawinski, P., Spiewak, M., Seggewiss, H., and Bilinska, Z.T. (2014). Does p.Q247X in *TRIM63* cause human hypertrophic cardiomyopathy? Circ. Res. *114*, e2–e5.
11. Lunter, G., and Goodson, M. (2011). Stampy: A statistical algorithm for sensitive and fast mapping of Illumina sequence reads. Genome Res. *21*, 936–939.
12. Rimmer, A., Phan, H., Mathieson, I., Iqbal, Z., Twigg, S.R.F., Wilkie, A.O.M., McVean, G., Lunter, G.; and WGS500 Consortium (2014). Integrating mapping-, assembly- and haplotype-based approaches for calling variants in clinical sequencing applications. Nat. Genet. *46*, 912–918.
13. Carss, K.J., Arno, G., Erwood, M., Stephens, J., Sanchis-Juan, A., Hull, S., Megy, K., Grozeva, D., Dewhurst, E., Malka, S., et al.; NIHR-BioResource Rare Diseases Consortium (2017). Comprehensive rare variant analysis via whole-genome sequencing to determine the molecular pathology of inherited retinal disease. Am. J. Hum. Genet. *100*, 75–90.
14. Calabrese, C., Simone, D., Diroma, M.A., Santorsola, M., Guttà, C., Gasparre, G., Picardi, E., Pesole, G., and Attimonelli, M. (2014). MToolBox: A highly automated pipeline for heteroplasmy annotation and prioritization analysis of human mitochondrial variants in high-throughput sequencing. Bioinformatics *30*, 3115–3117.
15. Richards, S., Aziz, N., Bale, S., Bick, D., Das, S., Gastier-Foster, J., Grody, W.W., Hegde, M., Lyon, E., Spector, E., et al.; ACMG Laboratory Quality Assurance Committee (2015). Standards and guidelines for the interpretation of sequence variants: A joint consensus recommendation of the American College of Medical Genetics and Genomics and the Association for Molecular Pathology. Genet. Med. *17*, 405–424.
16. Kircher, M., Witten, D.M., Jain, P., O'Roak, B.J., Cooper, G.M., and Shendure, J. (2014). A general framework for estimating the relative pathogenicity of human genetic variants. Nat. Genet. *46*, 310–315.
17. Lee, C., Kalmar, L., Xue, B., Tompa, P., Daughdrill, G.W., Uversky, V.N., and Han, K.H. (2014). Contribution of proline to the pre-structuring tendency of transient helical secondary structure elements in intrinsically disordered proteins. Biochim. Biophys. Acta *1840*, 993–1003.
18. Thermann, R., Neu-Yilik, G., Deters, A., Frede, U., Wehr, K., Hagemeier, C., Hentze, M.W., and Kulozik, A.E. (1998). Binary specification of nonsense codons by splicing and cytoplasmic translation. EMBO J. *17*, 3484–3494.
19. Kremer, L.S., Bader, D.M., Mertes, C., Kopajtich, R., Pichler, G., Iuso, A., Haack, T.B., Graf, E., Schwarzmayr, T., Terrile, C., et al. (2017). Genetic diagnosis of Mendelian disorders via RNA sequencing. Nat. Commun. *8*, 15824.
20. Pesta, D., and Gnaiger, E. (2012). High-resolution respirometry: OXPHOS protocols for human cells and permeabilized fibers from small biopsies of human muscle. Methods Mol. Biol. *810*, 25–58.
21. Oláhová, M., Hardy, S.A., Hall, J., Yarham, J.W., Haack, T.B., Wilson, W.C., Alston, C.L., He, L., Aznauryan, E., Brown, R.M., et al. (2015). *LRPPRC* mutations cause early-onset multisystem mitochondrial disease outside of the French-Canadian population. Brain *138*, 3503–3519.
22. Zerbetto, E., Vergani, L., and Dabbeni-Sala, F. (1997). Quantification of muscle mitochondrial oxidative phosphorylation enzymes via histochemical staining of blue native polyacrylamide gels. Electrophoresis *18*, 2059–2064.
23. Neuhoff, V., Arold, N., Taube, D., and Ehrhardt, W. (1988). Improved staining of proteins in polyacrylamide gels including isoelectric focusing gels with clear background at nanogram sensitivity using Coomassie Brilliant Blue G-250 and R-250. Electrophoresis *9*, 255–262.

24. Stroud, D.A., Maher, M.J., Lindau, C., Vögtle, F.N., Frazier, A.E., Surgenor, E., Mountford, H., Singh, A.P., Bonas, M., Oeljeklaus, S., et al. (2015). COA6 is a mitochondrial complex IV assembly factor critical for biogenesis of mtDNA-encoded COX2. Hum. Mol. Genet. *24*, 5404–5415.
25. Wirth, C., Brandt, U., Hunte, C., and Zickermann, V. (2016). Structure and function of mitochondrial complex I. Biochim. Biophys. Acta *1857*, 902–914.
26. Fuhrmann, D.C., Wittig, I., Dröse, S., Schmid, T., Dehne, N., and Brüne, B. (2018). Degradation of the mitochondrial complex I assembly factor TMEM126B under chronic hypoxia. Cell. Mol. Life Sci. *75*, 3051–3067.
27. Heide, H., Bleier, L., Steger, M., Ackermann, J., Dröse, S., Schwamb, B., Zörnig, M., Reichert, A.S., Koch, I., Wittig, I., and Brandt, U. (2012). Complexome profiling identifies TMEM126B as a component of the mitochondrial complex I assembly complex. Cell Metab. *16*, 538–549.
28. Angerer, H., Radermacher, M., Mańkowska, M., Steger, M., Zwicker, K., Heide, H., Wittig, I., Brandt, U., and Zickermann, V. (2014). The LYR protein subunit NB4M/NDUFA6 of mitochondrial complex I anchors an acyl carrier protein and is essential for catalytic activity. Proc. Natl. Acad. Sci. USA *111*, 5207–5212.
29. Ogilvie, I., Kennaway, N.G., and Shoubridge, E.A. (2005). A molecular chaperone for mitochondrial complex I assembly is mutated in a progressive encephalopathy. J. Clin. Invest. *115*, 2784–2792.
30. Mckenzie, M., and Ryan, M.T. (2010). Assembly factors of human mitochondrial complex I and their defects in disease. IUBMB Life *62*, 497–502.
31. Lazarou, M., McKenzie, M., Ohtake, A., Thorburn, D.R., and Ryan, M.T. (2007). Analysis of the assembly profiles for mitochondrial- and nuclear-DNA-encoded subunits into complex I. Mol. Cell. Biol. *27*, 4228–4237.
32. Stroud, D.A., Surgenor, E.E., Formosa, L.E., Reljic, B., Frazier, A.E., Dibley, M.G., Osellame, L.D., Stait, T., Beilharz, T.H., Thorburn, D.R., et al. (2016). Accessory subunits are integral for assembly and function of human mitochondrial complex I. Nature *538*, 123–126.
33. Wu, M., Gu, J., Guo, R., Huang, Y., and Yang, M. (2016). Structure of mammalian respiratory supercomplex $I_1III_2IV_1$. Cell *167*, 1598–1609.e10.
34. Alston, C.L., Compton, A.G., Formosa, L.E., Strecker, V., Oláhová, M., Haack, T.B., Smet, J., Stouffs, K., Diakumis, P., Ciara, E., et al. (2016). Biallelic mutations in *TMEM126B* cause severe complex I deficiency with a variable clinical phenotype. Am. J. Hum. Genet. *99*, 217–227.
35. Sánchez-Caballero, L., Ruzzenente, B., Bianchi, L., Assouline, Z., Barcia, G., Metodiev, M.D., Rio, M., Funalot, B., van den Brand, M.A., Guerrero-Castillo, S., et al. (2016). Mutations in complex I assembly factor *TMEM126B* result in muscle weakness and isolated complex I deficiency. Am. J. Hum. Genet. *99*, 208–216.
36. Punzi, G., Porcelli, V., Ruggiu, M., Hossain, M.F., Menga, A., Scarcia, P., Castegna, A., Gorgoglione, R., Pierri, C.L., Laera, L., et al. (2018). *SLC25A10* biallelic mutations in intractable epileptic encephalopathy with complex I deficiency. Hum. Mol. Genet. *27*, 499–504.
37. Schottmann, G., Picker-Minh, S., Schwarz, J.M., Gill, E., Rodenburg, R.J.T., Stenzel, W., Kaindl, A.M., and Schuelke, M. (2017). Recessive mutation in *EXOSC3* associates with mitochondrial dysfunction and pontocerebellar hypoplasia. Mitochondrion *37*, 46–54.
38. Haack, T.B., Haberberger, B., Frisch, E.M., Wieland, T., Iuso, A., Gorza, M., Strecker, V., Graf, E., Mayr, J.A., Herberg, U., et al. (2012). Molecular diagnosis in mitochondrial complex I deficiency using exome sequencing. J. Med. Genet. *49*, 277–283.
39. Swalwell, H., Kirby, D.M., Blakely, E.L., Mitchell, A., Salemi, R., Sugiana, C., Compton, A.G., Tucker, E.J., Ke, B.X., Lamont, P.J., et al. (2011). Respiratory chain complex I deficiency caused by mitochondrial DNA mutations. Eur. J. Hum. Genet. *19*, 769–775.

The American Journal of Human Genetics, Volume *103*

Supplemental Data

Bi-allelic Mutations in *NDUFA6* Establish Its Role in Early-Onset Isolated Mitochondrial Complex I Deficiency

Charlotte L. Alston, Juliana Heidler, Marris G. Dibley, Laura S. Kremer, Lucie S. Taylor, Carl Fratter, Courtney E. French, Ruth I.C. Glasgow, René G. Feichtinger, Isabelle Delon, Alistair T. Pagnamenta, Helen Dolling, Hugh Lemonde, Neil Aiton, Alf Bjørnstad, Lisa Henneke, Jutta Gärtner, Holger Thiele, Katerina Tauchmannova, Gerardine Quaghebeur, Josef Houstek, Wolfgang Sperl, F. Lucy Raymond, Holger Prokisch, Johannes A. Mayr, Robert McFarland, Joanna Poulton, Michael T. Ryan, Ilka Wittig, Marco Henneke, and Robert W. Taylor

Supplemental figures

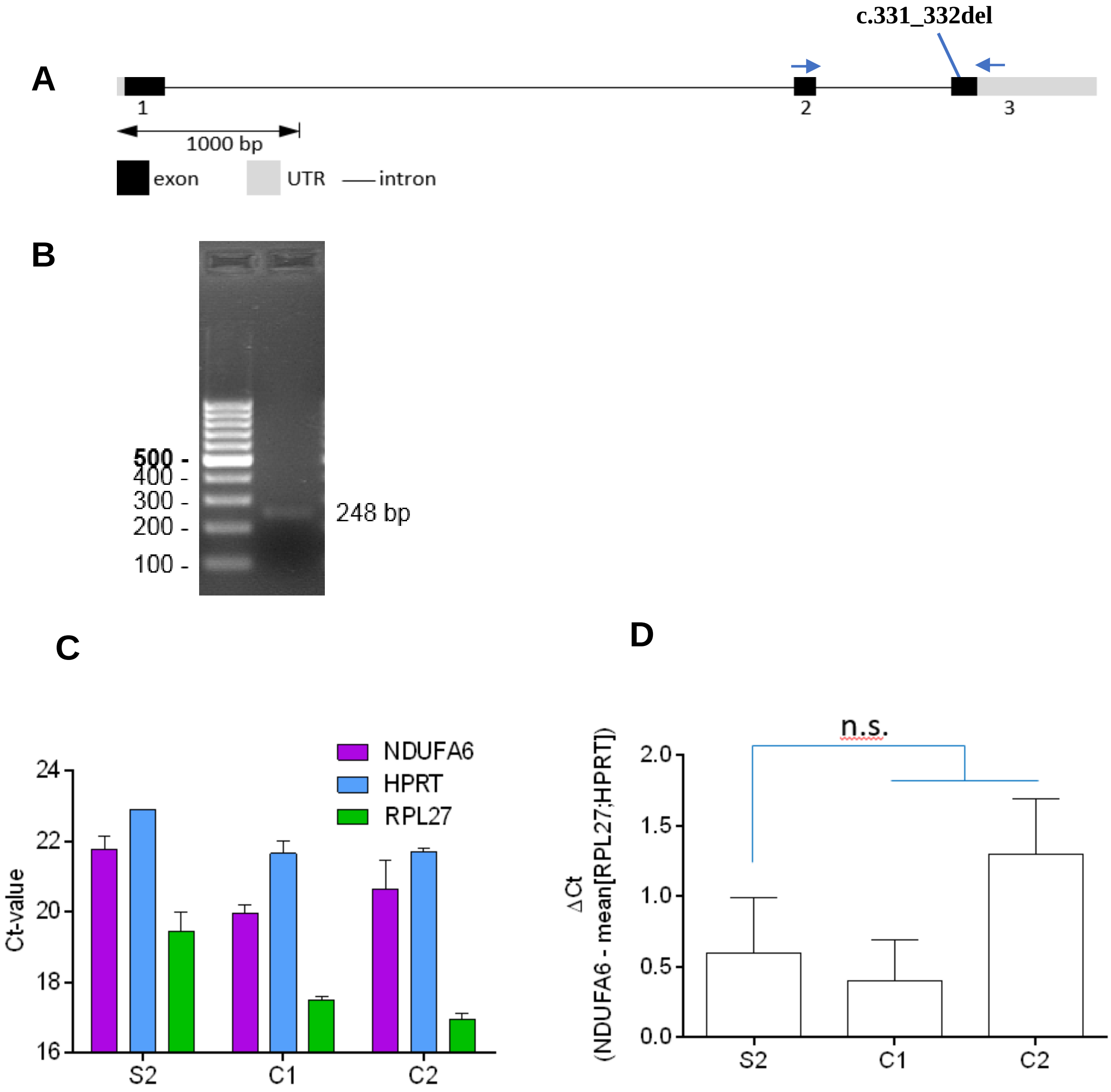

Figure S1. Normal amount of *NDUFA6* cDNA from fibroblasts in subject 2 (S2). A cDNA was prepared by reverse transcription using oligo-dT primers. Quantitative PCR of NDUFA6 was performed with a forward primer (5'-TGAAAATGGGACGGGATAAA-3') and reverse primer binding downstream of the frame-shift variant c.331_332del (5'-CAACGTGCATCTTTCCACTG-3'). Arrows indicate primer binding locations. **B** Agarose gel electrophoresis showed normal size (248 bp) of the *NDUFA6* PCR product. **C** Cycle threshold values for the NDUFA6 transcripts of S2 and two controls (C1, C2) are shown. **D** No difference in expression of NDUFA6 was seen between S2 and controls (using Student’s unpaired T-test). Error Bars indicate the standard deviation, the experiments were performed in triplicates.

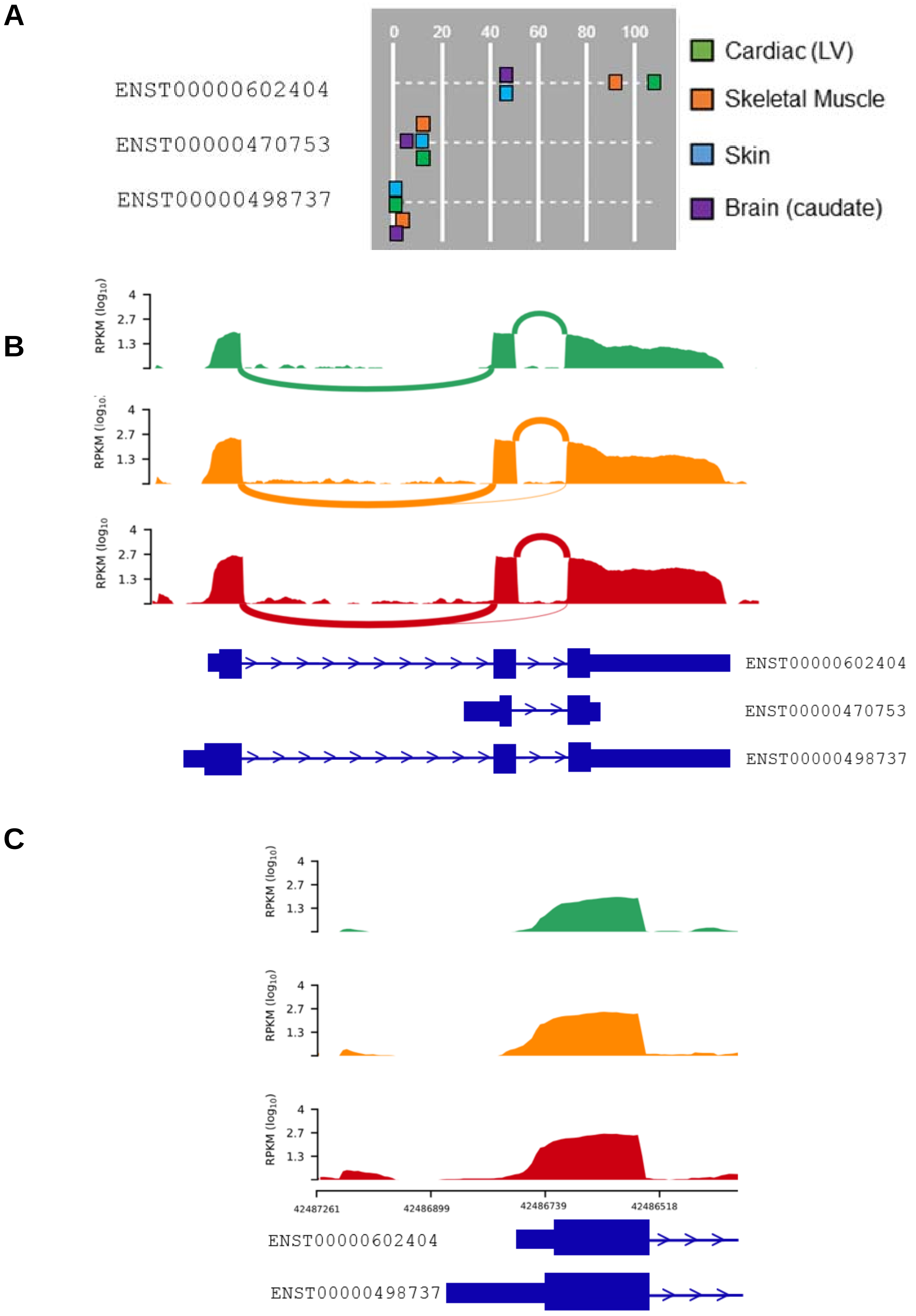

Figure S2. Alternative NDUFA6 transcripts and transcriptomics. A. Comparative RNAseq expression data obtained from the GTex repository supports highest expression of

the ENST00000602404 transcript in cardiac muscle, skeletal muscle, brain and skin (fibroblasts), with considerably lower expression of the alternative transcripts ENST00000498737 and ENST00000470753 (data correct at 04.04.2018). **B.** In-house RNAseq data supports the GTex data, providing further evidence that the ENST00000602404 transcript is predominantly expressed in skeletal muscle (green), cardiac muscle (red) and fibroblasts (orange). **C.** RNA expression at the 5'UTR corroborates ENST00000602404 as the predominant transcript in skeletal muscle (green), cardiac muscle (red) and fibroblasts (orange), with the transcription start site corresponding to that of ENST00000602404 and not ENST00000498737.

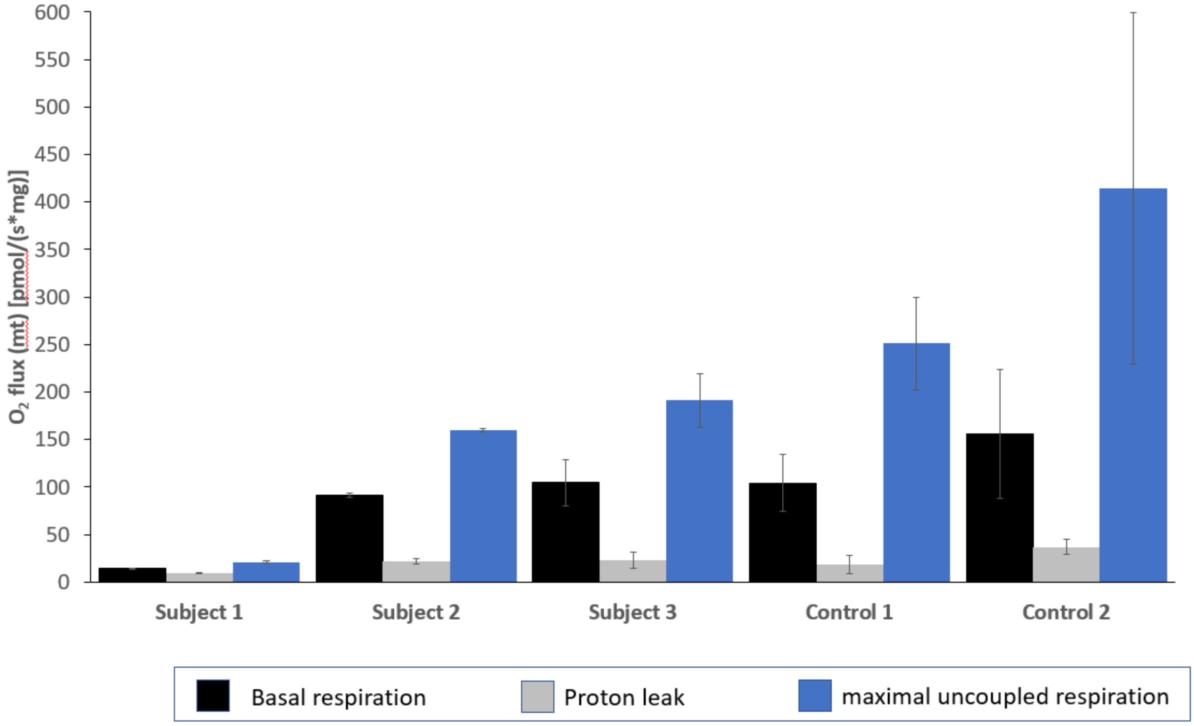

Figure S3. High resolution respirometry of Subject fibroblast cell lines.

Respiration of subject-derived fibroblasts compared to control fibroblasts shows basal respiration (**black**), oligomycin-inhibited (proton leak) respiration (**grey**) and maximum uncoupled respiration (**blue**) corrected for non-mitochondrial oxygen consumption. Data are mean ± SD from at least 2 experiments.

A B

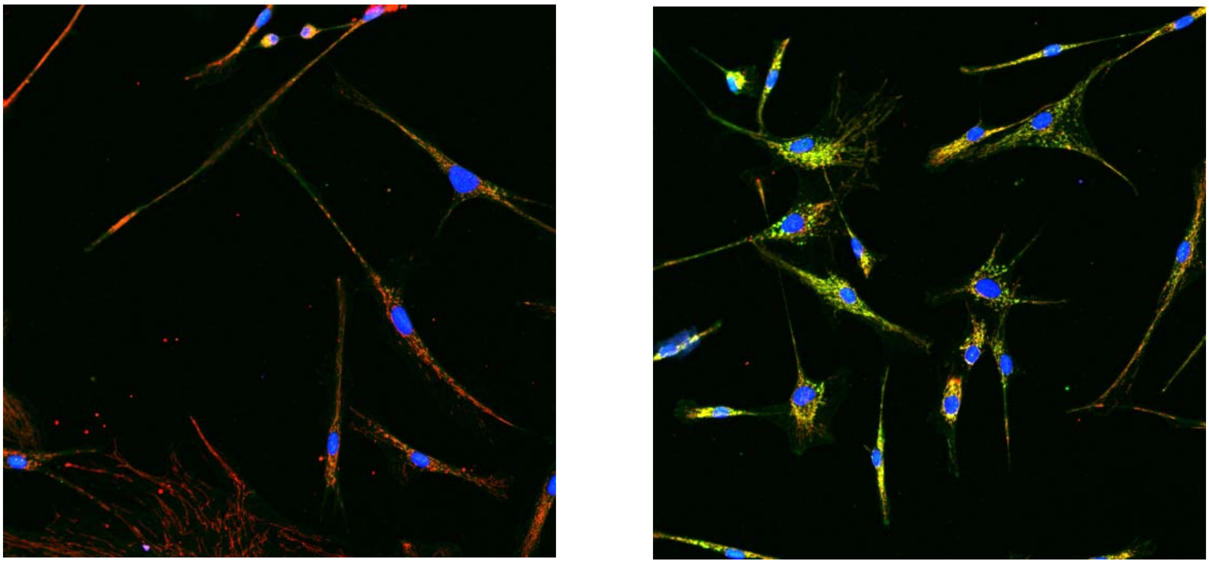

Figure S4: Cultured skin fibroblasts from individual S2 express a complex I defect
Immunofluorescence staining of fibroblasts obtained from individual S2 and a control was performed as reported[1] using the following primary antibodies: mouse monoclonal anti-NDUFS4 antibody detected in green (1:200; ab137064, Abcam, Cambridge, UK) and rabbit polyclonal anti-VDAC1 detected in red (1:400; ab15895, Abcam, Cambridge, UK) clearly demonstrating strong staining of the NDUFS4 (complex I) protein in the control (**B**) and absence in the patient cell line in the merged image (**A**). Scale bar = 50 µm.

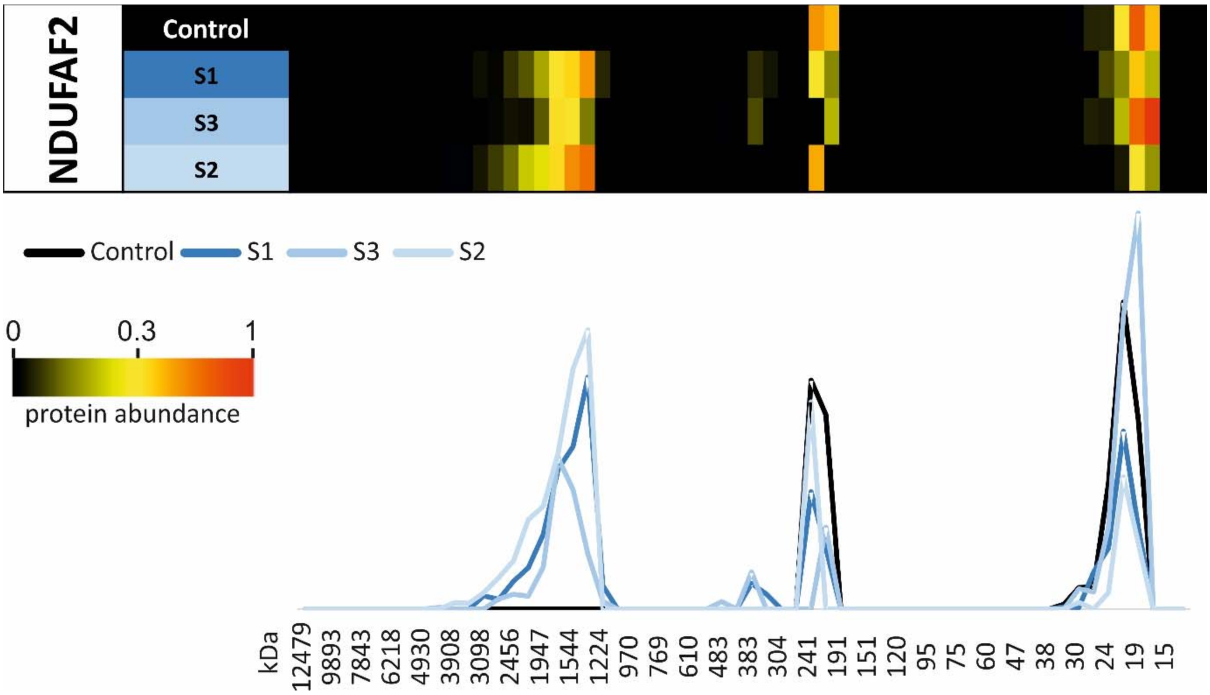

Figure S5. Retention of NDUFAF2 (mimitin) in Subjects' complexome profiles.
Excerpt of complexome profiling data reveals NDUFAF2 is detectable at significant levels in the subcomplex state of supercomplexes in Subject cells. The molecular weight (approximately 1.6MDa) is consistent with the N-module lacking respirasome, CI/CIII$_2$/CIV. There is no evidence of NDUFAF2 in the supercomplex state of control fibroblast cell lines, suggesting that in subjects harbouring pathogenic *NDUFA6* variants, NDUFAF2 fails to dissociate from the assembly intermediate and enables supercomplex formation despite the absent N-module.

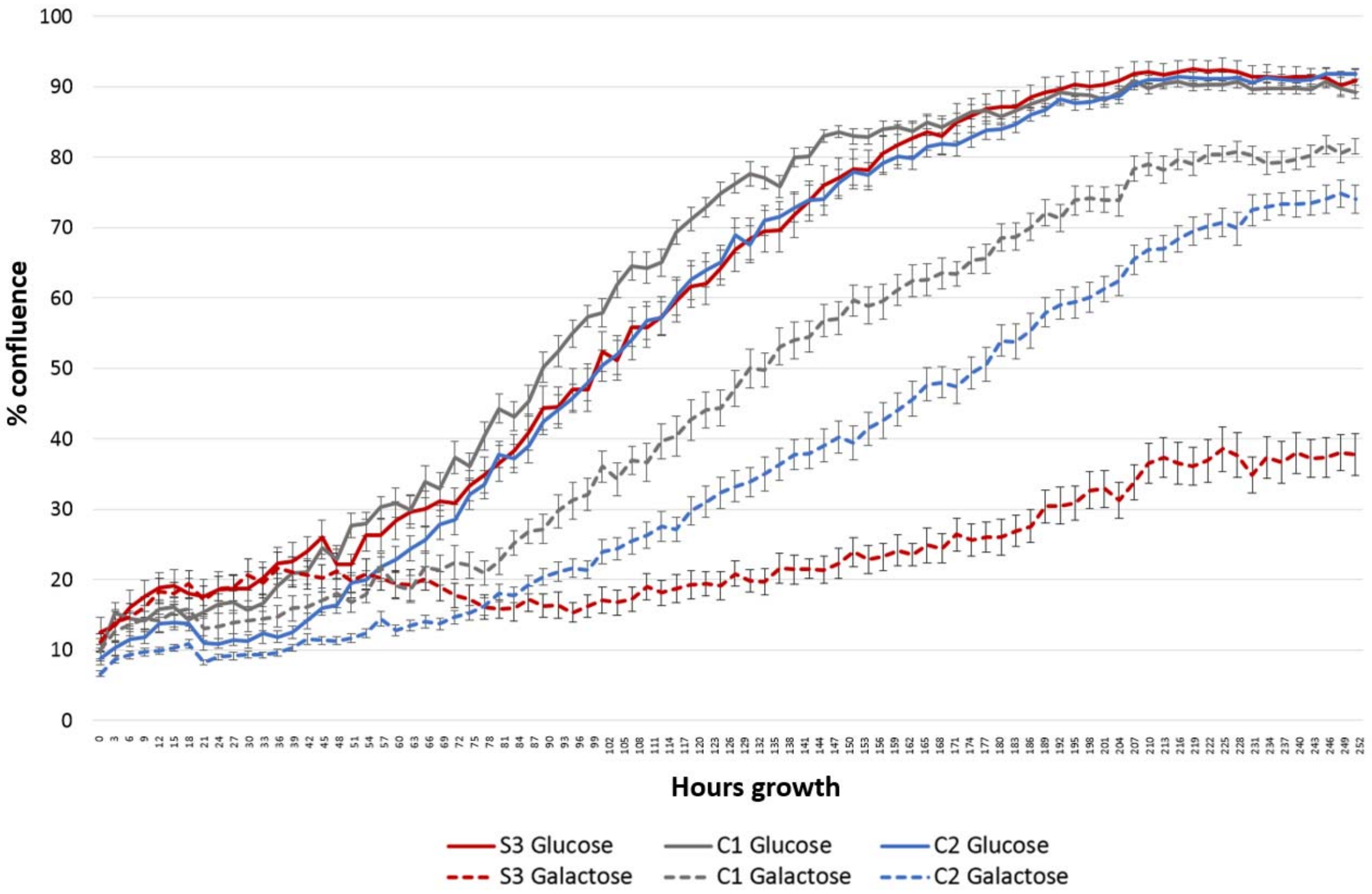

Figure S6. Glucose vs Galactose growth curves for fibroblasts from Subject 3.

Fibroblasts of Subject 3 and two controls were seeded, at 4,000 cells per well, into a 96 well plate (32 wells per cell line) in high glucose cell media containing 4.5 mg/ml glucose. After 15 hours, all media was removed and cells were washed with phosphate buffered saline. High glucose media was then returned to 16 wells per cell line, while the remaining 16 wells were switched to media containing 0.9 mg/ml galactose. Cell confluence was measured every 3 hours using the Incucyte® Live Cell Analysis System for 10 days. The fibroblasts of S3 demonstrated a delayed recovery of cell growth and significantly decreased growth rate in galactose media when compared with controls consistent with defective oxidative phosphorylation.

Supplemental Table 1. Details of commercially-available primary antibodies used in this study.

Primary antibody	Source	Catalogue number
NDUFV1	Proteintech	11238-1-AP
NDUFA13	Abcam	ab110240
MT-ND1	gift from Dr Anne Lombes	n/a
NDUFB8	Abcam	ab110242
NDUFA9	Abcam	ab14713
SDHA	Abcam	ab14715
VDAC1	Abcam	ab14734
UQCRC2	Abcam	ab14745
MT-COI	Abcam	ab14705
ATP5F1A	Abcam	ab14748

Supplemental references

1. Ahting, U., Mayr, J.A., Vanlander, A.V., Hardy, S.A., Santra, S., Makowski, C., Alston, C.L., Zimmermann, F.A., Abela, L., Plecko, B., *et al.* (2015). Clinical, biochemical, and genetic spectrum of seven patients with *NFU1* deficiency. Frontiers in genetics **6**, 123.
